# Real-world evaluation of sodium-glucose co-transporter-2 inhibitors and dipeptidyl peptidase-4 inhibitors for managing type 2 diabetes mellitus: a retrospective multi-ethnic cohort study

**DOI:** 10.1007/s40200-022-01004-4

**Published:** 2022-03-03

**Authors:** Louise Gek Huang Goh, Jiandong Sun, Benjamin Shao Kiat Ong, Daphne Khoo, Chee Fang Sum, Kwong Ng

**Affiliations:** 1 Agency for Care Effectiveness (ACE), Ministry of Health, 16 College Road, College of Medicine Building, Singapore, 169854 Singapore; 2 Diabetes Centre, Admiralty Medical Centre and Khoo Teck Puat Hospital, 90 Yishun Central, Singapore, 768828 Singapore

**Keywords:** SGLT2 inhibitors, DPP4 inhibitors, type 2 diabetes, real-world, cohort study, diabetes outcomes

## Abstract

**Abstract:**

**Purpose:**

Sodium-glucose co-transporter-2 (SGLT2) inhibitors and dipeptidyl peptidase-4 (DPP4) inhibitors are increasingly used as second-line therapies in patients with type 2 diabetes. The aim of this study was to assess the real-world effects of SGLT2 inhibitors in a multi-ethnic population in Singapore.

**Methods:**

This retrospective cohort study examined patients diagnosed with and treated for diabetes from the Ministry of Health’s administrative database. Differences in outcomes between treatment groups were assessed using Poisson regression. Demographics, clinical characteristics, previous diagnoses and hospitalisations, and diabetes medication history were used for propensity score matching. Subgroup analyses by ethnicity were performed. Effect size was estimated using risk ratios (RRs) with 95% confidence intervals (CIs).

**Results:**

Patients initiating SGLT2 inhibitors were more likely to achieve glycaemic control target than DPP4 inhibitor-treated patients (RR 1.09; 95% CI 1.04, 1.14). This was observed only in patients of Chinese ethnicity. A higher risk of diabetic ketoacidosis in SGLT2 inhibitor initiators was not observed. SGLT2 inhibitors were associated with reduced risk of hypoglycaemia (RR 0.69; 95% CI 0.59, 0.82) and urinary tract infection (RR 0.52; 95% CI 0.43, 0.63) but was not statistically significant for hypoglycaemia in Malay patients. Compared to DPP4 inhibitors, SGLT2 inhibitors were associated with 12% and 34% reduction in any-cause hospitalisation and all-cause mortality, respectively, potentially resulting in more than $50 million savings over 10 years.

**Conclusion:**

SGLT2 inhibitors were associated with improvements in glycaemic control, reduced risk of complications, and was well tolerated. Ethnicity also plays a role and should be considered in future studies.

## Background

Type 2 diabetes mellitus (T2DM) is a major concern worldwide and a main cause of death in most countries [1]. The International Diabetes Federation estimated that about 463 million adults had diabetes, with 4.2 million deaths due to diabetes in 2019 [2]. The Western Pacific region including Singapore had the highest number of deaths. In 2045, the number of people with diabetes is expected to increase to about 700 million. The prevalence of type 2 diabetes in Singapore adults aged 18 to 69 years will also double from 7.3% in 1990 to 15.0% in 2050 [3]. T2DM, if not well controlled, can further lead to complications like kidney failure, lower limb amputation, nerve damage, cardiovascular disease (CVD), loss of vision and severe disabilities [4–6]. In addition, Asian patients with T2DM tend to have an earlier onset compared to their Caucasian counterparts. Nearly one-fifth (18%) were first diagnosed before 40 years old with a mean age of 32.9 years [7], compared to 13% in the United States (US) population aged between 18 to 44 years [8]. This further increases the risk of T2DM complications with longer disease duration. Optimal glycaemic control is thus crucial for preventing or delaying the development and progression of these complications [9]. A glycaemic control target, haemoglobin A1c (HbA1c) of below 7% is considered reasonable for most adults to achieve and is used to identify patients with good control [10].

At present, the main classes of oral glucose-lowering agents registered in Singapore include biguanides, sulfonylureas, sodium-glucose co-transporter-2 (SGLT2) inhibitors, dipeptidyl peptidase-4 (DPP4) inhibitors, meglitinides, thiazolidinediones and alpha-glucosidase inhibitors [10]. Metformin, a biguanide, is recommended as first-line therapy due to its long-term efficacy and safety data [10]. It is well tolerated with a low risk of hypoglycaemia and weight gain [9]. However, it is often insufficient as a monotherapy to manage the condition as disease progresses, and multiple agents are required to control blood glucose [11]. While sulfonylureas are considered a suitable add-on therapy, they may increase the risk of hypoglycaemia. Newer drug classes like SGLT2 inhibitors and DPP4 inhibitors are increasingly being used as second-line hypoglycaemic agents when sulfonylureas are not tolerated or when hypoglycaemia is a concern [12, 13]. Of note, DPP4 inhibitors can be used regardless of level of kidney function as long as the dosage is adjusted according to estimated glomerular filtration rate (eGFR) [14]. In contrast, SGLT2 inhibitors are contraindicated in those with moderate to severe kidney impairment [15, 16].

Three SGLT2 inhibitors (dapagliflozin, empagliflozin and canagliflozin) have been registered in Singapore since 2014. Their use is encouraged over DPP4 inhibitors given the availability of outcomes data and favourable cost-effectiveness [10, 17]. It remains unclear if the use of SGLT2 inhibitors in the local context is associated with the desired outcomes shown in clinical trials, while real-world studies comparing SGLT2 inhibitors with DPP4 inhibitors were mainly done in the western countries. To date, only a small local retrospective cohort study of 57 patients compared the effects of canagliflozin and sitagliptin on glycaemic control [18]. Given ethnicity is a significant predictor of HbA1c levels, local evidence is needed to assess the real-world effectiveness of these newer drug classes in specific ethnic subgroups and the Singapore general population [19]. This national study aimed to compare the effects of SGLT2 inhibitors with DPP4 inhibitors on patient outcomes in an ethnically diverse Asian population using real-world evidence and further translate such differences into any potential healthcare cost savings.

## Methods

### Study design and data source

In this retrospective cohort study, the Ministry of Health (MOH)‘s administrative database containing national-level healthcare use data was accessed. It contained anonymised data from public hospitals and primary care clinics, with about 8 million diabetes prescription records up to 2018. The study population was a large representative sample of patients with T2DM who sought treatment in the public healthcare setting in Singapore. Information on demographics, disease diagnoses, prescription records and investigation results of these patients were studied. Ethics approval was not required as the intent of this study was to assess the effect of SGLT2 inhibitors on clinical outcomes for the purpose of improving routine clinical care.

Disease diagnoses were recorded using the International Classification of Diseases, Tenth Revision Australian Modification (ICD-10 AM) codes. All T2DM patients aged 30 years and above receiving SGLT2 inhibitors (dapagliflozin, empagliflozin and canagliflozin) or DPP4 inhibitors (linagliptin, sitagliptin, vildagliptin and saxagliptin) at public healthcare institutions were included in the analyses. Patients were included in the study if they had a diagnosis and treatment for diabetes. For individuals with non-specific diagnosis codes, patients with type 1 diabetes mellitus (T1DM) were differentiated and excluded based on age at diagnosis and treatment. Patients diagnosed at age less than 40 years and on insulin only were classified as T1DM. This approach had also been used by other studies in identifying patients with T1DM [20, 21]. Non-residents who were not routinely managed and followed up in Singapore, and patients with no information on age, gender, age below 30 years or had a death record were also excluded.

### Patient selection and baseline characteristics

T2DM patients newly initiating SGLT2 inhibitors or DPP4 inhibitors between January 2015 and December 2018 were included in this analysis. A washout period of one year was used to identify new users. The earliest prescription date was defined as the treatment initiation date. Patients were assigned to either SGLT2 inhibitor or DPP4 inhibitor-treated cohort dependent on the treatment they were initiated on. Those who had any prescriptions of studied drugs before the initiation date were excluded to restrict the cohorts to only new users. An intention to treat approach was used for the analysis where patients were followed from initiation of index treatment to observation of outcome or end of follow-up period (whichever was earlier).

Baseline characteristics were obtained for each patient during the one year before initiation. These variables included prescribing setting (public hospitals and primary care clinics), year of first prescription of SGLT2 inhibitors or DPP4 inhibitors, duration with diabetes, age, gender, ethnicity, resident status, body weight (in kilograms, kg), blood pressure (in mmHg), smoking status, subsidy status or socioeconomic status category, any hospitalisation, hospitalisation for DM complications [poor diabetes control (ICD-10 AM: E1*65), diabetic kidney complications (E1*2*), insulin resistance (E1*72), hypoglycaemia (E1*64), retinopathy (E1*3*), neuropathy (E1*4*), peripheral angiopathy (E1*5*) and foot ulcer (E1*73)], co-morbidities (CVD, cancer, hypertensive disease and hyperlipidaemia disease), glycaemic control rate i.e. HbA1c (%), eGFR (mL/min/1.73m^2^) and DM medication history (number of oral DM medications, and use of metformin, sulfonylureas, acarbose and insulin). The differences in baseline characteristics were compared using Student t-test for continuous variables and Pearson’s chi-squared test for categorical variables. Standardised differences were also used to compare baseline characteristics between the treatment cohorts.

### Definition of outcomes and statistical analyses

The efficacy and safety of SGLT2 inhibitors and DPP4 inhibitors were assessed as classes of drugs since the individual drugs within the drug classes have the same mechanism of action with comparable clinical effectiveness and safety [17]. The outcomes measured were glycaemic control during 91–365 days after initiation as patients were typically followed up every three months, and any-cause, cause-specific hospitalisations, and all-cause death during 31–365 days after initiation. The HbA1c result nearest to the treatment initiation date was used as the baseline while the result closest to the date of 365 days after initiation was used as the post-treatment data [12]. Patients with missing HbA1c results during the follow-up period were excluded from the analysis. Cause-specific hospitalisations with these admission diagnoses were included in the analyses: diabetic ketoacidosis (DKA) (ICD-10 AM: E1*1* e.g. E1111 T2DM with ketoacidosis, without coma), primary T2DM (E11-E14), primary T2DM with kidney complications (E1*2*), incipient diabetic nephropathy (E1*21), hypoglycaemia (E1*64), CVD (I00-I99) and heart failure (HF) (I50*) as a secondary outcome with previous HF hospitalisation included as a co-variate, and urinary tract infection (UTI) (N10, N12, N136, N151, N159, N30, N300, N308, N309, N390). Only the first hospitalisation of each outcome was included in the analysis. Subgroup analyses by ethnicity (Chinese, Malay and Indian) were also performed to assess potential differential effect of SGLT2 inhibitors on patient outcomes.

Each patient in the SGLT2 inhibitor-treated cohort was matched with a patient from the DPP4 inhibitor-treated cohort with the nearest propensity score (PS), to account for differences in baseline characteristics and enable a more homogeneous comparison. Patients were matched 1:1 on PS which was derived from a logistic model using all co-variates described. This was similarly done in the subgroup analysis where PS was derived and matched within each ethnic group. The balance in the two cohorts was assessed using standardised differences (a value less than 0.1 indicates negligible differences) [22, 23]. Finally, modified Poisson regression models [24] were also used to estimate risk ratios (RRs) and 95% confidence intervals (CIs) for the matched SGLT2 inhibitor and DPP4 inhibitor-treated cohorts with and without adjustment. P-values lower than 0.05 were considered to be statistically significant. All analyses were performed using Stata version 16.

To derive the healthcare costs saved due to improvements in patient outcomes associated with SGLT2 inhibitor use, a Markov model was used to estimate the cumulative number of deaths and hospitalisations avoided and quantify the costs saved over 10 years. Cost savings were quantified by multiplying the difference in hospitalisation rates between the treatment cohorts by the number of patients on SGLT2 inhibitors and mean T2DM hospitalisation cost (assumed to remain unchanged). This difference in hospitalisation rate was applied across the years, with prevalent cases rolled over from the preceding year plus the incident cases in the current year. In addition, adjustments were made on the projected patient numbers excluding those due to deaths. These analyses were performed using Microsoft Excel.

## Results

### Baseline demographics and clinical characteristics

**Fig. 1:**
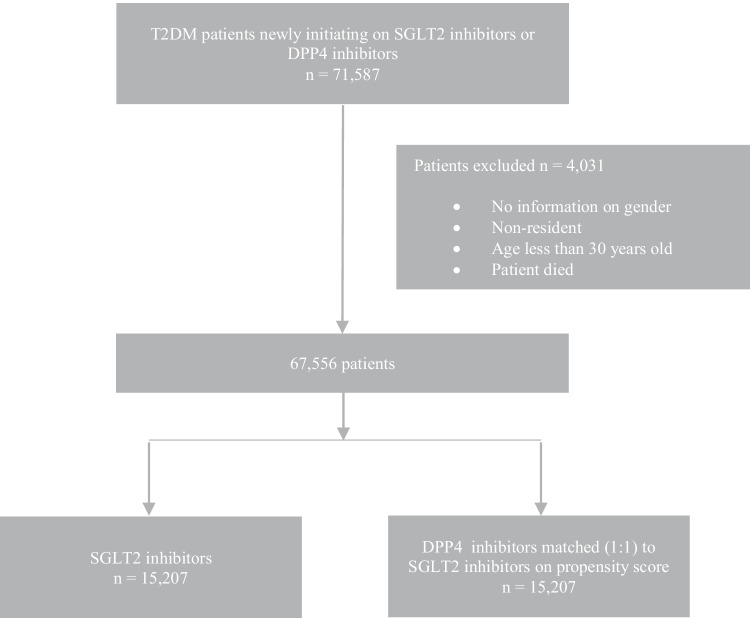
Selection of study population for outcomes measured 31–365 days after initiation

**Fig. 2:**
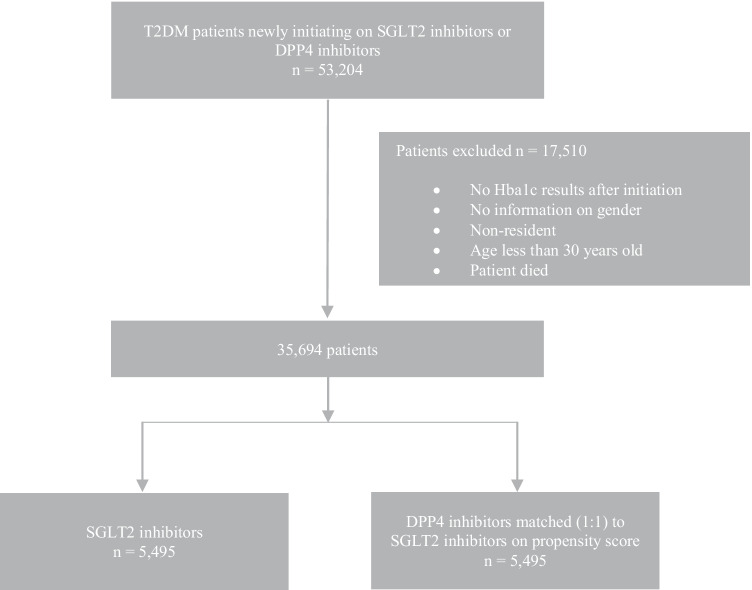
Selection of study population for outcomes measured 91–365 days after initiation

There were 71,587 eligible patients with outcomes measured 31–365 days after initiation. After excluding those below 30 years, non-residents, with missing information on gender or age, and those with a death record within 30 days of treatment initiation, 67,556 patients remained. Most patients were initiators of DPP4 inhibitors (about 77%). Before matching, the two cohorts differed significantly on most baseline characteristics, with absolute standardised difference greater than 0.1. Patients in the SGLT2 inhibitor-treated cohort were younger compared to the DPP4 inhibitor-treated cohort (mean age 56 years vs. 63 years). There were more patients in the SGLT2 inhibitor-treated cohort with body weight 80 kg and above (21% vs. 15%). In addition, more patients on SGLT2 inhibitors had disease duration of less than 5 years (31% vs. 21%) and fewer DM complications prior to treatment initiation (e.g. 2% vs. 14% for DM-kidney complications). However, more patients on SGLT2 inhibitors were using multiple oral drugs (39% vs. 29% on two drugs), metformin (64% vs. 44%) and insulin (21% vs. 19%) than DPP4 inhibitor-treated cohort. After PS matching, 15,207 comparable patients remained in each cohort with outcomes measured 31–365 days after initiation (Table 1 and Fig. 1). The results on the 35,694 eligible patients with outcomes measured 91–365 days after initiation and 5495 comparable patients in each cohort after matching are provided in Appendix Table 5 and Fig. 2. The baseline characteristics of patients from different ethnic groups are also reported in Appendix Tables 6, 7, 8, 9, 10 and 11. The characteristics were well balanced after matching between the two cohorts.

**Table 1:** Comparison of baseline characteristics in two treatment cohorts before and after PS matching

Variables	Unmatched cohorts			Matched cohorts		
	DPP4 inhibitors	SGLT2 inhibitors	d	DPP4 inhibitors	SGLT2 inhibitors	d
	(n = 52,349)	(n = 15,207)		(n = 15,207)	(n = 15,207)	
Age (years), mean ± SD	62.9 ± 11.6	56.3 ± 10.2	0.110	57.3 ± 10.8	56.3 ± 10.2	0.016
	n(%)	n(%)		n(%)	n(%)	
Setting of initiation						
Hospitals	22,669 (43.3%)	5535 (36.4%)	0.140	5519 (36.3%)	5535 (36.4%)	0.002
Primary care clinics	29,680 (56.7%)	9672 (63.6%)	0.141	9688 (63.7%)	9672 (63.6%)	0.002
Year of initiation						
2015	6933 (13.2%)	406 (2.7%)	0.400	347 (2.3%)	406 (2.7%)	0.055
2016	12,425 (23.7%)	734 (4.8%)	0.560	721 (4.7%)	734 (4.8%)	0.004
2017	16,240 (31.0%)	5515 (36.3%)	0.111	5960 (39.2%)	5515 (36.3%)	0.060
2018	16,751 (32.0%)	8552 (56.2%)	0.503	8179 (53.8%)	8552 (56.2%)	0.049
Gender (male)	27,175 (51.9%)	8361 (55.0%)	0.062	8265 (54.4%)	8361 (55.0%)	0.013
Ethnicity						
Chinese	33,071 (63.2%)	9199 (60.5%)	0.060	9204 (60.5%)	9199 (60.5%)	0.001
Indian	7326 (14.0%)	2461 (16.2%)	0.061	2450 (16.1%)	2461 (16.2%)	0.002
Malay	7297 (13.9%)	2087 (13.7%)	0.010	2124 (14.0%)	2087 (13.7%)	0.007
Others	4655 (8.9%)	1460 (9.6%)	0.025	1429 (9.4%)	1460 (9.6%)	0.007
Residence						
SC	50,326 (96.1%)	14,423 (94.8%)	0.060	14,445 (95.0%)	14,423 (94.8%)	0.007
PR	2023 (3.9%)	784 (5.2%)	0.063	762 (5.0%)	784 (5.2%)	0.007
SES category						
Maximum subsidy	23,281 (44.5%)	4751 (31.2%)	0.280	4842 (31.8%)	4751 (31.2%)	0.013
Some subsidy	1059 (2.0%)	436 (2.9%)	0.055	403 (2.7%)	436 (2.9%)	0.013
Minimum subsidy	1338 (2.6%)	500 (3.3%)	0.043	506 (3.3%)	500 (3.3%)	0.002
NA	26,671 (51.0%)	9520 (62.6%)	0.237	9456 (62.2%)	9520 (62.6%)	0.009
Weight (kilograms)						
<65	15,673 (29.9%)	3334 (21.9%)	0.180	3424 (22.5%)	3334 (21.9%)	0.014
65–79	13,110 (25.0%)	3729 (24.5%)	0.010	3841 (25.3%)	3729 (24.5%)	0.017
≥80	7692 (14.7%)	3178 (20.9%)	0.163	2942 (19.4%)	3178 (20.9%)	0.039
NA	15,874 (30.3%)	4966 (32.7%)	0.050	5000 (32.9%)	4966 (32.7%)	0.005
Cigarette smoking (number of cigarettes per day)						
Non-smoker	19,379 (37.0%)	5046 (33.2%)	0.080	5092 (33.5%)	5046 (33.2%)	0.006
1–9	1585 (3.0%)	522 (3.4%)	0.023	506 (3.3%)	522 (3.4%)	0.006
≥10	3092 (5.9%)	1003 (6.6%)	0.028	983 (6.5%)	1003 (6.6%)	0.006
NA	28,293 (54.1%)	8636 (56.8%)	0.055	8626 (56.7%)	8636 (56.8%)	0.001
Diastolic BP (mmHg)						
<65	11,053 (21.1%)	2638 (17.4%)	0.100	2683 (17.6%)	2638 (17.4%)	0.008
65–89	27,929 (53.4%)	8101 (53.3%)	0.002	8034 (52.8%)	8101 (53.3%)	0.009
≥90	1882 (3.6%)	582 (3.8%)	0.012	599 (3.9%)	582 (3.8%)	0.006
NA	11,485 (21.9%)	3886 (25.6%)	0.085	3891 (25.6%)	3886 (25.6%)	0.001
Systolic BP (mmHg)						
<130	16,336 (31.2%)	4644 (30.5%)	0.020	4575 (30.1%)	4644 (30.5%)	0.010
130–139	11,575 (22.1%)	3393 (22.3%)	0.005	3432 (22.6%)	3393 (22.3%)	0.006
≥140	12,953 (24.7%)	3284 (21.6%)	0.070	3309 (21.8%)	3284 (21.6%)	0.004
NA	11,485 (21.9%)	3886 (25.6%)	0.085	3891 (25.6%)	3886 (25.6%)	0.001
Duration with diabetes (years)						
0–4	10,897 (20.8%)	4773 (31.4%)	0.242	4473 (29.4%)	4773 (31.4%)	0.043
5–9	16,189 (30.9%)	4188 (27.5%)	0.080	4202 (27.6%)	4188 (27.5%)	0.002
≥10	24,482 (46.8%)	5837 (38.4%)	0.170	6151 (40.5%)	5837 (38.4%)	0.042
NA	781 (1.5%)	409 (2.7%)	0.084	381 (2.5%)	409 (2.7%)	0.011
Diagnoses for hospitalisation 1–365 days prior to initiation						
Any hospitalisation	18,783 (35.9%)	3130 (20.6%)	0.350	3207 (21.1%)	3130 (20.6%)	0.013
DM-kidney complications	7051 (13.5%)	368 (2.4%)	0.420	375 (2.5%)	368 (2.4%)	0.003
Retinopathy	2651 (5.1%)	513 (3.4%)	0.080	538 (3.5%)	513 (3.4%)	0.009
Neuropathy	802 (1.5%)	75 (0.5%)	0.100	70 (0.5%)	75 (0.5%)	0.004
Peripheral angiopathy	1285 (2.5%)	86 (0.6%)	0.160	79 (0.5%)	86 (0.6%)	0.007
Poor control	6409 (12.2%)	856 (5.6%)	0.230	894 (5.9%)	856 (5.6%)	0.011
Hypoglycaemia	3072 (5.9%)	173 (1.1%)	0.260	170 (1.1%)	173 (1.1%)	0.002
Insulin resistance	15,477 (29.6%)	2236 (14.7%)	0.360	2270 (14.9%)	2236 (14.7%)	0.006
Foot ulcer	1203 (2.3%)	124 (0.8%)	0.120	139 (0.9%)	124 (0.8%)	0.010
HbA1c (%)						
<7	4983 (9.5%)	1261 (8.3%)	0.040	1253 (8.2%)	1261 (8.3%)	0.002
7–8.9	19,638 (37.5%)	5264 (34.6%)	0.060	5328 (35.0%)	5264 (34.6%)	0.009
≥9	11,716 (22.4%)	3378 (22.2%)	0.004	3300 (21.7%)	3378 (22.2%)	0.012
NA	16,012 (30.6%)	5304 (34.9%)	0.092	5326 (35.0%)	5304 (34.9%)	0.003
eGFR (mL/min/1.73m^2^)						
<60	7499 (14.3%)	588 (3.9%)	0.370	526 (3.5%)	588 (3.9%)	0.022
60–89	6973 (13.3%)	1964 (12.9%)	0.010	2041 (13.4%)	1964 (12.9%)	0.015
≥90	7202 (13.8%)	2622 (17.2%)	0.096	2709 (17.8%)	2622 (17.2%)	0.015
NA	30,675 (58.6%)	10,033 (66.0%)	0.153	9931 (65.3%)	10,033 (66.0%)	0.014
Diagnoses in 3 years prior to initiation (co-morbid conditions)						
Any CVD	23,257 (44.4%)	4568 (30.0%)	0.300	4614 (30.3%)	4568 (30.0%)	0.007
Any cancer	1676 (3.2%)	197 (1.3%)	0.130	207 (1.4%)	197 (1.3%)	0.005
Hypertensive disease	21,286 (40.7%)	3859 (25.4%)	0.330	3935 (25.9%)	3859 (25.4%)	0.011
Hyperlipidaemia	19,579 (37.4%)	3727 (24.5%)	0.280	3766 (24.8%)	3727 (24.5%)	0.006
Medication history of DM drugs 1–365 days prior to initiation						
Number of oral DM drugs						
No records	12,738 (24.3%)	3324 (21.9%)	0.060	3672 (24.2%)	3324 (21.9%)	0.054
1	20,987 (40.1%)	5277 (34.7%)	0.110	5001 (32.9%)	5277 (34.7%)	0.038
2	15,281 (29.2%)	5861 (38.5%)	0.199	5785 (38.0%)	5861 (38.5%)	0.010
≥3	3343 (6.4%)	745 (4.9%)	0.070	749 (4.9%)	745 (4.9%)	0.001
MET	23,075 (44.1%)	9711 (63.9%)	0.405	9199 (60.5%)	9711 (63.9%)	0.070
SU	33,520 (64.0%)	8497 (55.9%)	0.170	8612 (56.6%)	8497 (55.9%)	0.015
Acarbose	3690 (7.1%)	661 (4.4%)	0.120	688 (4.5%)	661 (4.4%)	0.008
Insulin	9971 (19.1%)	3112 (20.5%)	0.035	2966 (19.5%)	3112 (20.5%)	0.024

DPP4: dipeptidyl peptidase 4; SGLT2: sodium-glucose co-transporter 2; d: standardised difference; SD: standard deviation; SC: Singapore citizen; PR: Singapore permanent resident; SES: socioeconomic status; NA: not applicable; BP: blood pressure; DM: diabetes mellitus; HbA1c: haemoglobin A1c; eGFR: estimated glomerular filtration rate; CVD: cardiovascular disease; MET: metformin; SU: sulfonylureas

### Glycaemic control

In the matched cohort, SGLT2 inhibitor initiation was associated with a significantly lower mean HbA1c than those initiated on DPP4 inhibitors (7.54% vs. 7.68%, p < 0.001). A higher proportion of patients also achieved good glycaemic control, HbA1c below 7% (40.8% SGLT2 vs. 37.5% DPP4) with a RR of 1.09 (95% CI 1.04, 1.14). They were also less likely to report poor glycaemic control with HbA1c above 8% (RR 0.88; 95% CI 0.83, 0.94). The difference between treatment groups was however not statistically significant in patients with HbA1c between 7% and 8% in the overall cohort (Table 2). Similar results were observed only in patients of Chinese ethnicity while no significant difference were observed in patients of Malay and Indian ethnicity except lower risk of having HbA1c between 7% and 8% in Indian patients on SGLT2 inhibitors.

**Table 2:** RR and associated 95% CIs for glycaemic control in two treatment cohorts after treatment initiation

Outcomes, n(%)	DPP4 inhibitors	SGLT2 inhibitors	Unadjusted RR (95% CI)	Adjusted RR (95% CI)^
***All patients (5495 matched patients from each treatment cohort)***				
HbA1c < 7%	2062 (37.5%)	2240 (40.8%)	1.09 (1.04, 1.14)	**1.09 (1.04, 1.14)**
HbA1c 7–8%	1740 (31.7%)	1761 (32.1%)	1.01 (0.96, 1.07)	1.01 (0.96, 1.07)
HbA1c > 8%	1693 (30.8%)	1494 (27.2%)	0.88 (0.83, 0.94)	**0.88 (0.83, 0.94)**
***Chinese (3365 matched patients from each treatment cohort)***				
HbA1c < 7%	1264 (37.6%)	1382 (41.1%)	1.09 (1.03, 1.16)	**1.09 (1.03, 1.16)**
HbA1c 7–8%	1138 (33.8%)	1150 (34.2%)	1.01 (0.95, 1.08)	1.01 (0.95, 1.08)
HbA1c > 8%	963 (28.6%)	833 (24.8%)	0.87 (0.80, 0.94)	**0.87 (0.80, 0.94)**
***Indian (905 matched patients from each treatment cohort)***				
HbA1c < 7%	318 (35.1%)	357 (39.5%)	1.12 (1.00, 1.27)	1.11 (0.99, 1.25)
HbA1c 7–8%	294 (32.5%)	254 (28.1%)	0.86 (0.75, 0.99)	**0.86 (0.75, 0.99)**
HbA1c > 8%	293 (32.4%)	294 (32.5%)	1.00 (0.88, 1.15)	1.00 (0.88, 1.14)
***Malay (745 matched patients from each treatment cohort)***				
HbA1c < 7%	280 (37.6%)	303 (40.7%)	1.08 (0.95, 1.23)	1.07 (0.94, 1.21)
HbA1c 7–8%	222 (29.8%)	218 (29.3%)	0.98 (0.84, 1.15)	0.98 (0.84, 1.15)
HbA1c > 8%	243 (32.6%)	224 (30.1%)	0.92 (0.79, 1.07)	0.92 (0.80, 1.07)

^Adjusted for baseline HbA1c and year of initiation (for Indian and Malay patients)

HbA1c: haemoglobin A1c; DPP4: dipeptidyl peptidase 4; SGLT2: sodium-glucose co-transporter 2; RR: risk ratio; CI: confidence interval

### Safety outcomes

In terms of safety outcomes, patients initiating SGLT2 inhibitors were not at higher risk of experiencing DKA compared to DPP4 inhibitors (Table 3). This was similarly observed for risk of DKA hospitalisation with length of stay seven days and longer. The risks of hospitalisation for hypoglycaemia (RR 0.69; 95% CI 0.59, 0.82) were reduced with SGLT2 inhibitors and there was no increased risk of UTI hospitalisations (RR 0.52; 95% CI 0.43, 0.63). These results were also observed across all ethnic groups except in patients of Malay ethnicity. The risk of DKA was significantly reduced in this ethnic group while no significant difference was observed in the occurrence of hypoglycaemia hospitalisations with SGLT2 inhibitor initiation.

**Table 3:** RR and associated 95% CIs for DKA, hypoglycaemia and UTI in two treatment cohorts after treatment initiation

Outcomes, n (%)	DPP4 inhibitors	SGLT2 inhibitors	RR (95%CI)
***All patients (15,207 matched patients from each treatment cohort)***			
DKA	108 (0.7%)	83 (0.6%)	0.77 (0.58, 1.02)
DKA hospitalisation with length of stay ≥7 days	47 (0.3%)	44 (0.3%)	0.94 (0.62, 1.41)
Hospitalised for hypoglycaemia	347 (2.3%)	241 (1.6%)	**0.69 (0.59, 0.82)**
Hospitalised for UTI	332 (2.2%)	173 (1.1%)	**0.52 (0.43, 0.63)**
***Chinese (9199 matched patients from each treatment cohort)***			
DKA	55 (0.6%)	51 (0.6%)	0.93 (0.63, 1.36)
DKA hospitalisation with length of stay ≥7 days	27 (0.3%)	25 (0.3%)	0.93 (0.54, 1.59)
Hospitalised for hypoglycaemia	189 (2.1%)	124 (1.4%)	**0.66 (0.52, 0.82)**
Hospitalised for UTI	166 (1.8%)	95 (1.0%)	**0.57 (0.45, 0.74)**
***Indian (2461 matched patients from each treatment cohort)***			
DKA	22 (0.9%)	11 (0.5%)	0.50 (0.24, 1.03)
DKA hospitalisation with length of stay ≥7 days	10 (0.4%)	6 (0.2%)	0.60 (0.22, 1.65)
Hospitalised for hypoglycaemia	94 (3.8%)	48 (2.0%)	**0.51 (0.36, 0.72)**
Hospitalised for UTI	74 (3.0%)	39 (1.6%)	**0.53 (0.36, 0.77)**
***Malay (2087 matched patients from each treatment cohort)***			
DKA	30 (1.4%)	15 (0.7%)	**0.50 (0.27, 0.93)**
DKA hospitalisation with length of stay ≥7 days	12 (0.6%)	10 (0.5%)	0.83 (0.36, 1.92)
Hospitalised for hypoglycaemia	49 (2.4%)	44 (2.1%)	0.90 (0.60, 1.34)
Hospitalised for UTI	59 (2.8%)	24 (1.2%)	**0.41 (0.25, 0.65)**

DPP4: dipeptidyl peptidase 4; SGLT2: sodium-glucose co-transporter 2; RR: risk ratio; CI: confidence interval; DKA: diabetic ketoacidosis; UTI: urinary tract infection

### Hospitalisations and deaths

In addition, SGLT2 inhibitors were associated with fewer hospitalisations and deaths up to one-year post-initiation compared to DPP4 inhibitors (Table 4). Any-cause and cause-specific hospitalisations ranged between 12% (any hospitalisation) and 72% (hospitalised for DM-related kidney complications) lower in the SGLT2 inhibitor-treated cohort compared to the DPP4 inhibitor-treated cohort. However, there was no difference in risk of diabetic nephropathy (except in patients of Indian ethnicity) and CVD hospitalisation between the treatment cohorts. Lower risk of all-cause mortality was observed among patients initiating SGLT2 inhibitors versus DPP4 inhibitors, with RR of 0.66 (95% CI 0.51, 0.85). Circulatory system diseases, neoplasms and respiratory diseases were the most common causes of death. The lower risk of hospitalisations and deaths associated with SGLT2 inhibitors were similarly observed in patients of Chinese and Indian ethnicity (except risk of all-cause death was not statistically significant). In patients of Malay ethnicity, only hospitalisation risk for DM-related kidney complications was significantly reduced in patients on SGLT2 inhibitors compared to those on DPP4 inhibitors.

**Table 4:** RR and associated 95% CIs for hospitalisations and deaths in two treatment cohorts after treatment initiation

Outcomes, n(%)	DPP4 inhibitors	SGLT2 inhibitors	RR (95%CI)
***All patients (15,207 matched patients from each treatment cohort)***			
Any hospitalisation	2830 (18.6%)	2489 (16.4%)	**0.88 (0.84, 0.92)**
Hospitalised for DM (principal diagnosis)	546 (3.6%)	336 (2.2%)	**0.62 (0.54, 0.70)**
Hospitalised for DM-related kidney complications	156 (1.0%)	44 (0.3%)	**0.28 (0.20, 0.39)**
Hospitalised for diabetic nephropathy	34 (0.2%)	37 (0.2%)	1.09 (0.68, 1.73)
Hospitalised for CVD	534 (3.5%)	570 (3.8%)	1.07 (0.95, 1.20)
Hospitalised for HF	211 (1.4%)	164 (1.1%)	**0.78 (0.63, 0.95)**
All-cause death	151 (1.0%)	100 (0.7%)	**0.66 (0.51, 0.85)**
***Chinese (9199 matched patients from each treatment cohort)***			
Any hospitalisation	1467 (16.0%)	1263 (13.7%)	**0.86 (0.80, 0.92)**
Hospitalised for DM (principal diagnosis)	280 (3.0%)	145 (1.6%)	**0.52 (0.42, 0.63)**
Hospitalised for DM-related kidney complications	92 (1.0%)	20 (0.2%)	**0.22 (0.13, 0.35)**
Hospitalised for diabetic nephropathy	10 (0.1%)	18 (0.2%)	1.80 (0.83, 3.90)
Hospitalised for CVD	299 (3.3%)	293 (3.2%)	0.98 (0.84, 1.15)
Hospitalised for HF	124 (1.4%)	76 (0.8%)	**0.61 (0.46, 0.81)**
All-cause death	87 (1.0%)	56 (0.6%)	**0.64 (0.46, 0.90)**
***Indian (2461 matched patients from each treatment cohort)***			
Any hospitalisation	594 (24.1%)	521 (21.2%)	**0.88 (0.79, 0.97)**
Hospitalised for DM (principal diagnosis)	114 (4.6%)	65 (2.6%)	**0.57 (0.42, 0.77)**
Hospitalised for DM-related kidney complications	27 (1.1%)	9 (0.4%)	**0.33 (0.16, 0.71)**
Hospitalised for diabetic nephropathy	15 (0.6%)	5 (0.2%)	**0.33 (0.12, 0.92)**
Hospitalised for CVD	115 (4.7%)	126 (5.1%)	1.10 (0.86, 1.40)
Hospitalised for HF	47 (1.9%)	32 (1.3%)	0.68 (0.44, 1.06)
All-cause death	25 (1.0%)	17 (0.7%)	0.68 (0.37, 1.26)
***Malay (2087 matched patients from each treatment cohort)***			
Any hospitalisation	476 (22.8%)	441 (21.1%)	0.93 (0.83, 1.04)
Hospitalised for DM (principal diagnosis)	101 (4.8%)	84 (4.0%)	0.83 (0.63, 1.10)
Hospitalised for DM-related kidney complications	26 (1.3%)	12 (0.6%)	**0.46 (0.23, 0.91)**
Hospitalised for diabetic nephropathy	5 (0.2%)	12 (0.6%)	2.40 (0.85, 6.80)
Hospitalised for CVD	82 (3.9%)	95 (4.6%)	1.16 (0.87, 1.55)
Hospitalised for HF	27 (1.3%)	40 (1.9%)	1.48 (0.91, 2.40)
All-cause death	32 (1.5%)	19 (0.9%)	0.59 (0.34, 1.04)

DPP4: dipeptidyl peptidase 4; SGLT2: sodium-glucose co-transporter 2; RR: risk ratio; CI: confidence interval; DM: diabetes mellitus; CVD: cardiovascular disease; HF: heart failure

In the secondary analysis on hospitalisations for HF, patients on SGLT2 inhibitors were less likely to be hospitalised compared to DPP4 inhibitor initiators (RR 0.78; 95% CI 0.63, 0.95) (Table 4). Among patients of Chinese ethnicity, a lower risk of HF hospitalisation was also observed in those initiating SGLT2 inhibitors compared to DPP4 inhibitors. There were no significant differences observed in patients of Malay or Indian ethnicity.

### Healthcare savings

These benefits associated with SGLT2 inhibitors versus DPP4 inhibitors would lead to about 1261 deaths avoided and 8691 fewer hospitalisations. This contributes to more than $50 million saved over 10 years.

## Discussion

This is the first national real-world study in Singapore that evaluated the potential impact of ethnicity on the effects of SGLT2 inhibitors and DPP4 inhibitors. PS matching was performed to balance baseline characteristics between the treatment groups to minimise bias. In addition, improvements in patient outcomes associated with SGLT2 inhibitor initiation was also translated to healthcare cost savings to the system.

Our findings are consistent with other real-world studies and clinical trials showing SGLT2 inhibitor initiation to be associated with a higher likelihood of achieving HbA1c targets compared to DPP4 inhibitor initiation (40.8% vs. 37.5%). Locally, a single-centre retrospective cohort study of 57 patients also reported that patients on canagliflozin were more likely to attain HbA1c levels below 7% than patients in the sitagliptin group (13.6% vs. 8.6%) at 24-week follow-up [18]. Another prospective Canadian registry study assessing outcomes associated with canagliflozin observed that more patients achieved HbA1c below 7% over time, reaching 38.8% by 12 months [25] which is similar to our findings of 40.8% up to one year follow-up. Similar findings were reported in real-world studies conducted in the US [26, 27]. In addition to canagliflozin, dapagliflozin also showed greater reductions in HbA1c than other oral antidiabetic drugs such as DPP4 inhibitors, with more patients attaining target glycaemic control or reduction in the real-world setting [28–30]. SGLT2 inhibitors also showed better glycaemic control than DPP4 inhibitors in clinical trials [31, 32]. A meta-analysis comprising 25 randomised controlled trials (RCTs) observed no statistically significant difference between the treatment groups but there was substantial heterogeneity across studies (I^2^ = 62%) [33].

As expected, the relative efficacy of treatments differed across ethnic groups. Although SGLT2 inhibitor use increased the likelihood of achieving target glycaemic control in patients of Chinese ethnicity, this was not observed in patients of Malay and Indian ethnicity. This is consistent with the literature that diabetes control is more optimal among the Chinese compared to Malays and Indians [34], thus highlighting the need to consider ethnicity in diabetes management and when assessing clinical outcomes. It is also important to realise that ethnicity is affected by genetic and environmental factors such as body fat distribution, adipose tissue function, differences in insulin secretion levels and insulin sensitivity, health beliefs and dietary habits [34, 35], forming a complex interplay of risk factors.

In terms of safety outcomes, the literature was mixed, with some studies reporting increased DKA risk with SGLT2 inhibitors and other studies reporting no increase. Our study did not observe a higher risk of hospitalisation for DKA with SGLT2 inhibitors. Similarly, another nationwide retrospective cohort study in Korea did not observe an increase in DKA risk in the SGLT2 inhibitor-treated group [hazard ratio (HR) 0.956; 95% CI 0.581, 1.572; p = 0.996] after PS matching [13]. The risk of DKA was also not higher in the SGLT2 inhibitor-treated group in a meta-analysis consisting of 81 trials, with Mantel-Haenszel odds ratio (OR) of 1.14 (95% CI 0.45, 2.88; p = 0.78) [36]. Two other meta-analyses [37, 38], the Empagliflozin Cardiovascular Outcome Event Trial in Type 2 Diabetes Mellitus Patients – Removing Excess Glucose (EMPA-REG OUTCOME) trial [39] and Canagliflozin Cardiovascular Assessment Study (CANVAS) programme [40] also reported similar results. On the other hand, a retrospective observational study in the US (HR 2.2; 95% CI 1.4, 3.6) and a cohort study on Scandinavian countries (HR 2.14; 95% CI 1.17, 4.09) found treatment with SGLT2 inhibitors to be associated with higher DKA risk than DPP4 inhibitors with PS matching [41, 42]. Clinicians may need to continue monitoring patients who are initially starting SGLT2 inhibitors, in particular, euglycaemic DKA which can be easily missed due to normal glucose levels [43–45] or when there are symptoms such as nausea and vomiting which may indicate ketoacidosis [46].

Hypoglycaemia results in our study are also consistent with those in the published literature. The risk of hospitalisations for hypoglycaemia was 31% lower in patients initiating SGLT2 inhibitors compared to DPP4 inhibitors in our study. This effect was similarly observed in the ethnic subgroups but was not statistically significant in patients of Malay ethnicity. A meta-analysis of nine RCTs also reported lower risk of hypoglycaemia with SGLT2 inhibitors (OR 0.48; 95% CI 0.28, 0.82; p = 0.008) [38]. This was also observed in real-world studies with patients receiving dapagliflozin reporting lower rates of hypoglycaemia than other oral drugs (0.6% vs. 1.3%) [28] and decreased risk of hypoglycaemia with SGLT2 inhibitors (HR 0.76; 95% CI 0.65, 0.90; p = 0.001) [47]. A systematic review comprising 25 RCTs (RR, 0.99; 95% CI 0.78, 1.26, p = 0.92) [33] and an additional RCT (4.0% vs. 3.4%) [31] found the risk or incidence of hypoglycaemic events to be similar between users of SGLT2 inhibitors and DPP4 inhibitors.

In addition, we observed that SGLT2 inhibitors did not increase the risk of UTI hospitalisations compared to DPP4 inhibitors in the overall cohort and across all ethnic groups. This is consistent with a large US cohort study of 123,752 matched patients on SGLT2 inhibitors and DPP4 inhibitors which also found a lower risk of UTI hospitalisations (HR 0.68; 95% CI 0.54, 0.87) [48]. Two meta-analyses did not report an increased risk of severe or non-severe UTI events with SGLT2 inhibitors [49, 50]. Another observational study in Australia similarly did not find a higher risk of UTI infections in SGLT2 inhibitor initiators (HR 0.90; 95% CI 0.66, 1.24) [51]. Other studies also reported similar UTI rates between treatment groups [31, 37, 52, 53] while a pooled analysis (OR 1.15; 95% CI 1.00, 1.33; p = 0.047) [38] and a retrospective cohort study in Korea (HR 1.05; 95% CI 1.00, 1.11; p = 0.047) [54] reported increased risk of UTIs with SGLT2 inhibitors which was borderline significant.

Our study also found that SGLT2 inhibitors reduced the risk of hospitalisations (except for CVD hospitalisations and hospitalisations for diabetic nephropathy) and all-cause death compared to DPP4 inhibitors. Other real-world studies also showed SGLT2 inhibitors were associated with a lower risk of all-cause death compared with other diabetes drugs (HR 0.51; 95% CI 0.37, 0.70; p < 0.001) [55]. Furthermore, this finding was consistent across countries, ranging from 25% in Singapore to 68% reduced risk in Australia. The lower risk of death was attenuated when restricted to first new-user and using intention to treat approach (HR 0.65; 95% CI 0.60, 0.71; p < 0.001) [55], similar to our study findings of 34% reduced risk of death in the SGLT2 inhibitor-treated cohort. Other observational studies [42, 56–58], clinical trials such as EMPA-REG OUTCOME trial [39] and CANVAS programme [40], and a meta-analysis [38] also reported a lower risk of all-cause death with SGLT2 inhibitors. A real-world study in Israel also reported reduced risk of hospitalisations (OR 0.662; 95% CI 0.564, 0.776; p < 0.001) in patients initiating SGLT2 inhibitors compared with DPP4 inhibitors up to 24 weeks and its effects were similarly observed in the matched populations (OR 0.731; 95% CI 0.603, 0.885; p = 0.001) [58]. As expected, the magnitude of decreased hospitalisation risk varied across ethnic groups with patients of Chinese ethnicity reporting greater reductions in hospitalisation and death risk than other ethnic groups in our study. This again highlights the importance of including ethnicity when assessing the impact of treatments on patient outcomes.

Although no significant differences were observed for CVD hospitalisations, SGLT2 inhibitor-treated patients were 22% less likely to be hospitalised for HF than DPP4 inhibitor-treated patients in our study. This is similarly observed in other retrospective observational studies in Korea (HR 0.66; 95% CI 0.58, 0.75; p < 0.001) [59] and US (HR 0.68; 95% CI 0.54, 0.86; p = 0.001) [60]. A network meta-analysis study of 58 trials also reported reduced HF events with SGLT2 inhibitors (HR 0.55; 95% CI 0.46, 0.67; I^2^ = 19%) [61]. Our findings are also consistent with those from the observational Comparative Effectiveness of Cardiovascular Outcomes in New Users of Sodium-Glucose Cotransporter-2 Inhibitors (CVD-REAL) 2 study comprising patients from six countries including Singapore. SGLT2 inhibitors were associated with 26% lower risk of HF hospitalisation than other oral and injectable glucose-lowering drugs (HR 0.74; 95% CI 0.69, 0.80) [55]. However, statistically significant reduction was not observed in patients from Singapore (HR 0.58; 95% CI 0.34, 1.00) likely due to the small sample size (n = 2222) [55]. The CVD-REAL Nordic study also observed a reduced risk of hospitalisations for HF with SGLT2 inhibitors compared to other diabetes drugs (HR 0.70; 95% CI 0.61, 0.81; p < 0.0001) [47]. In a later CVD-REAL Nordic study comparing dapagliflozin and DPP4 inhibitors, similar findings on hospitalisation for HF were reported (HR 0.69; 95% CI 0.57, 0.84; p < 0.001) [56]. A Scandinavian register based cohort study also found significant differences in HF events favouring SGLT2 inhibitors over DPP4 inhibitors (HR 0.66; 95% CI 0.53, 0.81) [42].

Our study also estimated that the reduced risk of hospitalisations associated with SGLT2 inhibitors would translate to cumulative savings of more than $50 million and 1261 deaths avoided over 10 years. Although the use of newer drugs such as SGLT2 inhibitors to improve glycaemic control would increase spending, these costs were offset by savings in the longer term from lower rates of co-morbidities [62].

One of the strengths of our study is the inclusion of a large and representative sample of ethnically and clinically diverse patients with T2DM seeking treatments in Singapore. In addition, PS matching was performed to balance baseline characteristics of patients between treatment groups and to minimise bias when assessing treatment effect [63]. Several variables were also used in the identification of T2DM patients such as age at diagnosis and treatment in addition to diagnosis codes. Thus the risk of misclassification for T2DM was low considering our study findings are also consistent with those reported in published real-world studies and clinical trials. There are however some limitations with using prescribing data. Prescribing data does not reflect actual ingestion and adherence to therapy but prescriptions indicated as cancelled or discontinued were excluded from the analyses, to capture medication use more accurately. Residual confounding may still remain after PS matching. Future studies with larger sample sizes or longer follow-up period may be required to further assess the effect of SGLT2 inhibitors by ethnicity on outcomes such as diabetic nephropathy. Possible switching between SGLT2 inhibitors and DPP4 inhibitors after treatment initiation was not accounted for. Finally, the benefits of SGLT2 inhibitors were potentially underestimated as reductions in body weight and blood pressure could not be assessed due to limitations of the database.

## Conclusions

In summary, the results of our study showed that SGLT2 inhibitors were associated with improvements in glycaemic control and reduced risk of hospitalisations and deaths in patients with T2DM managed in the public healthcare setting in Singapore, and were well tolerated. However, such benefits were mostly observed in patients of Chinese ethnicity. Therefore, future studies should consider ethnicity as a key factor in overall disease management and the risk of developing T2DM-related complications.

## Data Availability

Data will not be publicly shared but is available on reasonable request and if legal implications are fulfilled.

## Appendix A

Table 5

**Table 5:** Comparison of baseline characteristics in two treatment cohorts before and after PS matching for outcomes measured 91–365 days after initiation

Variables	Unmatched cohorts			Matched cohorts		
	DPP4 inhibitors	SGLT2 inhibitors	d	DPP4 inhibitors	SGLT2 inhibitors	d
	(n = 30,199)	(n = 5495)		(n = 5495)	(n = 5495)	
Age (years), mean ± SD	62.8 ± 11.3	56.7 ± 10.2	0.001	57.2 ± 11.0	56.7 ± 10.2	0.041
	n(%)	n(%)		n(%)	n(%)	
Setting of initiation						
Hospitals	12,270 (40.6%)	2162 (39.3%)	0.026	2192 (39.9%)	2162 (39.3%)	0.011
Primary care clinics	17,929 (59.4%)	3333 (60.7%)	0.026	3303 (60.1%)	3333 (60.7%)	0.011
Year of initiation						
2015	5495 (18.2%)	310 (5.6%)	0.395	242 (4.4%)	310 (5.6%)	0.057
2016	10,677 (35.4%)	634 (11.5%)	0.586	594 (10.8%)	634 (11.5%)	0.023
2017	10,180 (33.7%)	2798 (50.9%)	0.354	3002 (54.6%)	2798 (50.9%)	0.074
2018	3847 (12.7%)	1753 (31.9%)	0.473	1657 (30.2%)	1753 (31.9%)	0.038
Gender (male)	15,627 (51.8%)	3022 (55.0%)	0.065	3001 (54.6%)	3022 (55.0%)	0.008
Ethnicity						
Chinese	19,335 (64.0%)	3365 (61.2%)	0.058	3401 (61.9%)	3365 (61.2%)	0.013
Indian	4181 (13.8%)	905 (16.5%)	0.073	871 (15.9%)	905 (16.5%)	0.017
Malay	4065 (13.5%)	745 (13.6%)	0.003	748 (13.6%)	745 (13.6%)	0.001
Others	2618 (8.7%)	480 (8.7%)	0.002	475 (8.6%)	480 (8.7%)	0.004
Residence						
SC	29,181 (96.6%)	5264 (95.8%)	0.044	5276 (96.0%)	5264 (95.8%)	0.011
PR	1018 (3.4%)	231 (4.2%)	0.044	219 (4.0%)	231 (4.2%)	0.011
SES category						
Maximum subsidy	13,569 (44.9%)	1776 (32.3%)	0.261	1852 (33.7%)	1776 (32.3%)	0.029
Some subsidy	624 (2.1%)	166 (3.0%)	0.060	151 (2.8%)	166 (3.0%)	0.016
Minimum subsidy	723 (2.4%)	177 (3.2%)	0.050	171 (3.1%)	177 (3.2%)	0.006
NA	15,283 (50.6%)	3376 (61.4%)	0.220	3321 (60.4%)	3376 (61.4%)	0.020
Weight (kilograms)						
<65	9416 (31.2%)	1203 (21.9%)	0.212	1227 (22.3%)	1203 (21.9%)	0.011
65–79	8228 (27.3%)	1588 (28.9%)	0.037	1626 (29.6%)	1588 (28.9%)	0.015
≥80	4963 (16.4%)	1404 (25.6%)	0.225	1329 (24.2%)	1404 (25.6%)	0.031
NA	7592 (25.1%)	1300 (23.7%)	0.034	1313 (23.9%)	1300 (23.7%)	0.005
Cigarette smoking (number of cigarettes per day)						
Non-smoker	10,628 (35.2%)	1824 (33.2%)	0.042	1860 (33.9%)	1824 (33.2%)	0.014
1–9	1002 (3.3%)	215 (3.9%)	0.032	219 (4.0%)	215 (3.9%)	0.004
≥10	1894 (6.3%)	443 (8.1%)	0.069	418 (7.6%)	443 (8.1%)	0.017
NA	16,675 (55.2%)	3013 (54.8%)	0.008	2998 (54.6%)	3013 (54.8%)	0.005
Diastolic BP (mmHg)						
<65	6985 (23.1%)	1109 (20.2%)	0.072	1122 (20.4%)	1109 (20.2%)	0.006
65–89	17,294 (57.3%)	3326 (60.5%)	0.066	3277 (59.6%)	3326 (60.5%)	0.018
<90	1071 (3.6%)	225 (4.1%)	0.028	234 (4.3%)	225 (4.1%)	0.008
NA	4849 (16.1%)	835 (15.2%)	0.024	862 (15.7%)	835 (15.2%)	0.014
Systolic BP (mmHg)						
<130	10,135 (33.6%)	1879 (34.2%)	0.013	1875 (34.1%)	1879 (34.2%)	0.001
130–139	7177 (23.8%)	1387 (25.2%)	0.034	1351 (24.6%)	1387 (25.2%)	0.015
≥140	8038 (26.6%)	1394 (25.4%)	0.029	1407 (25.6%)	1394 (25.4%)	0.006
NA	4849 (16.1%)	835 (15.2%)	0.024	862 (15.7%)	835 (15.2%)	0.014
Duration with diabetes (years)						
0–4	5715 (18.9%)	1546 (28.1%)	0.218	1495 (27.2%)	1546 (28.1%)	0.021
5–9	10,577 (35.0%)	1578 (28.7%)	0.136	1539 (28.0%)	1578 (28.7%)	0.016
≥10	13,530 (44.8%)	2227 (40.5%)	0.086	2326 (42.3%)	2227 (40.5%)	0.037
NA	377 (1.3%)	144 (2.6%)	0.100	135 (2.5%)	144 (2.6%)	0.010
Diagnoses and complications 1–365 days prior to initiation						
Any hospitalisation	10,200 (33.8%)	1159 (21.1%)	0.287	1190 (21.7%)	1159 (21.1%)	0.014
DM-kidney complications	3774 (12.5%)	147 (2.7%)	0.377	159 (2.9%)	147 (2.7%)	0.013
Retinopathy	1455 (4.8%)	209 (3.8%)	0.050	204 (3.7%)	209 (3.8%)	0.005
Neuropathy	448 (1.5%)	33 (0.6%)	0.087	36 (0.7%)	33 (0.6%)	0.008
Peripheral angiopathy	643 (2.1%)	33 (0.6%)	0.132	35 (0.6%)	33 (0.6%)	0.005
Poor control	3606 (11.9%)	332 (6.0%)	0.207	342 (6.2%)	332 (6.0%)	0.008
Hypoglycaemia	1650 (5.5%)	71 (1.3%)	0.232	75 (1.4%)	71 (1.3%)	0.006
Insulin resistance	8537 (28.3%)	866 (15.8%)	0.305	886 (16.1%)	866 (15.8%)	0.010
Foot ulcer	596 (2.0%)	47 (0.9%)	0.094	47 (0.9%)	47 (0.9%)	0
HbA1c (%)						
<7	2257 (7.5%)	615 (11.2%)	0.128	598 (10.9%)	615 (11.2%)	0.010
7–8.9	11,414 (37.8%)	1953 (35.5%)	0.047	1930 (35.1%)	1953 (35.5%)	0.009
≥9	7848 (26.0%)	1286 (23.4%)	0.060	1307 (23.8%)	1286 (23.4%)	0.009
NA	8680 (28.7%)	1641 (29.9%)	0.025	1660 (30.2%)	1641 (29.9%)	0.008
eGFR (mL/min/1.73m^2^)						
<60	4429 (14.7%)	228 (4.2%)	0.366	235 (4.3%)	228 (4.2%)	0.006
60–89	3714 (12.3%)	712 (13.0%)	0.020	759 (13.8%)	712 (13.0%)	0.025
≥90	4189 (13.9%)	993 (18.1%)	0.115	1012 (18.4%)	993 (18.1%)	0.009
NA	17,867 (59.2%)	3562 (64.8%)	0.117	3489 (63.5%)	3562 (64.8%)	0.028
Diagnoses in 3 years prior to initiation (co-morbid conditions)						
Any CVD	13,266 (43.9%)	1771 (32.2%)	0.243	1806 (32.9%)	1771 (32.2%)	0.014
Any cancer	833 (2.8%)	86 (1.6%)	0.082	95 (1.7%)	86 (1.6%)	0.013
Hypertensive disease	12,131 (40.2%)	1521 (27.7%)	0.266	1563 (28.4%)	1521 (27.7%)	0.017
Hyperlipidaemia	11,260 (37.3%)	1446 (26.3%)	0.237	1477 (26.9%)	1446 (26.3%)	0.013
Medication history of DM drugs 1–365 days prior to initiation						
Number of oral DM drugs						
No records	5928 (19.6%)	948 (17.3%)	0.061	1014 (18.5%)	948 (17.3%)	0.031
1	12,315 (40.8%)	2113 (38.5%)	0.048	2080 (37.9%)	2113 (38.5%)	0.012
2	9406 (31.2%)	2099 (38.2%)	0.149	2073 (37.7%)	2099 (38.2%)	0.010
≥3	2550 (8.4%)	335 (6.1%)	0.090	328 (6.0%)	335 (6.1%)	0.005
MET	14,252 (47.2%)	3552 (64.6%)	0.357	3394 (61.8%)	3552 (64.6%)	0.060
SU	20,978 (69.5%)	3277 (59.6%)	0.207	3336 (60.7%)	3277 (59.6%)	0.022
Acarbose	2757 (9.1%)	301 (5.5%)	0.141	314 (5.7%)	301 (5.5%)	0.010
Insulin	6187 (20.5%)	1392 (25.3%)	0.115	1377 (25.1%)	1392 (25.3%)	0.006

DPP4: dipeptidyl peptidase 4; SGLT2: sodium-glucose co-transporter 2; d: standardised difference; SD: standard deviation; SC: Singapore citizen; PR: Singapore permanent resident; SES: socioeconomic status; NA: not applicable; BP: blood pressure; DM: diabetes mellitus; HbA1c: haemoglobin A1c; eGFR: estimated glomerular filtration rate; CVD: cardiovascular disease; MET: metformin; SU: sulfonylureas

Fig. 2

## Appendix B

Table 6

**Table 6:** Comparison of baseline characteristics in two treatment cohorts before and after PS matching for outcomes measured 31–365 days after initiation in patients of Chinese ethnicity

Variables	Unmatched cohorts			Matched cohorts		
	DPP4 inhibitors	SGLT2 inhibitors	d	DPP4 inhibitors	SGLT2 inhibitors	d
	(n = 33,071)	(n = 9199)		(n = 9199)	(n = 9199)	
Age (years), mean ± SD	64.7 ± 11.6	57.4 ± 10.6	0.119	58.5 ± 10.7	57.4 ± 10.6	0.019
	n(%)	n(%)		n(%)	n(%)	
Setting of initiation						
Hospitals	14,034 (42.4%)	3243 (35.3%)	0.148	3164 (34.4%)	3243 (35.3%)	0.018
care clinics	19,037 (57.6%)	5956 (64.8%)	0.148	6035 (65.6%)	5956 (64.8%)	0.018
Year of initiation						
2015	4365 (13.2%)	226 (2.5%)	0.408	216 (2.4%)	226 (2.5%)	0.016
2016	7768 (23.5%)	445 (4.8%)	0.555	415 (4.5%)	445 (4.8%)	0.016
2017	10,227 (30.9%)	3385 (36.8%)	0.124	3626 (39.4%)	3385 (36.8%)	0.054
2018	10,711 (32.4%)	5143 (55.9%)	0.488	4942 (53.7%)	5143 (55.9%)	0.044
Gender (male)	17,799 (53.8%)	5290 (57.5%)	0.074	5234 (56.9%)	5290 (57.5%)	0.012
Residence						
SC	32,250 (97.5%)	8896 (96.7%)	0.048	8913 (96.9%)	8896 (96.7%)	0.010
PR	821 (2.5%)	303 (3.3%)	0.048	286 (3.1%)	303 (3.3%)	0.010
SES category						
Maximum subsidy	14,255 (43.1%)	2616 (28.4%)	0.309	2634 (28.6%)	2616 (28.4%)	0.004
Some subsidy	565 (1.7%)	250 (2.7%)	0.069	221 (2.4%)	250 (2.7%)	0.020
Minimum subsidy	895 (2.7%)	315 (3.4%)	0.041	312 (3.4%)	315 (3.4%)	0.002
NA	17,356 (52.5%)	6018 (65.4%)	0.265	6032 (65.6%)	6018 (65.4%)	0.003
Weight (kilograms)						
<65	11,061 (33.5%)	2218 (24.1%)	0.207	2278 (24.8%)	2218 (24.1%)	0.015
65–79	8167 (24.7%)	2309 (25.1%)	0.009	2404 (26.1%)	2309 (25.1%)	0.024
≥80	4073 (12.3%)	1694 (18.4%)	0.170	1584 (17.2%)	1694 (18.4%)	0.031
NA	9770 (29.5%)	2978 (32.4%)	0.061	2933 (31.9%)	2978 (32.4%)	0.010
Cigarette smoking (number of cigarettes per day)						
Non-smoker	12,236 (37.0%)	2961 (32.2%)	0.101	3063 (33.3%)	2961 (32.2%)	0.024
1–9	915 (2.8%)	286 (3.1%)	0.020	278 (3.0%)	286 (3.1%)	0.005
≥10	2050 (6.2%)	618 (6.7%)	0.021	631 (6.9%)	618 (6.7%)	0.006
NA	17,870 (54.0%)	5334 (58.0%)	0.079	5227 (56.8%)	5334 (58.0%)	0.023
Diastolic BP (mmHg)						
<65	7330 (22.2%)	1637 (17.8%)	0.109	1671 (18.2%)	1637 (17.8%)	0.010
65–89	17,602 (53.2%)	4882 (53.1%)	0.003	4887 (53.1%)	4882 (53.1%)	0.001
≥90	1141 (3.5%)	360 (3.9%)	0.024	369 (4.0%)	360 (3.9%)	0.005
NA	6998 (21.2%)	2320 (25.2%)	0.096	2272 (24.7%)	2320 (25.2%)	0.012
Systolic BP (mmHg)						
<130	10,387 (31.4%)	2821 (30.7%)	0.016	2840 (30.9%)	2821 (30.7%)	0.004
130–139	7421 (22.4%)	2099 (22.8%)	0.009	2151 (23.4%)	2099 (22.8%)	0.013
≥140	8265 (25.0%)	1959 (21.3%)	0.088	1936 (21.1%)	1959 (21.3%)	0.006
NA	6998 (21.2%)	2320 (25.2%)	0.096	2272 (24.7%)	2320 (25.2%)	0.012
Duration with diabetes (years)						
0–4	6787 (20.5%)	2792 (30.4%)	0.227	2630 (28.6%)	2792 (30.4%)	0.039
5–9	10,027 (30.3%)	2568 (27.9%)	0.053	2517 (27.4%)	2568 (27.9%)	0.013
≥10	15,743 (47.6%)	3564 (38.7%)	0.180	3792 (41.2%)	3564 (38.7%)	0.051
NA	514 (1.6%)	275 (3.0%)	0.097	260 (2.8%)	275 (3.0%)	0.010
Diagnoses for hospitalisation 1–365 days prior to initiation						
Any hospitalisation	11,265 (34.1%)	1572 (17.1%)	0.397	1582 (17.2%)	1572 (17.1%)	0.003
DM-kidney complications	4325 (13.1%)	192 (2.1%)	0.424	164 (1.8%)	192 (2.1%)	0.023
Retinopathy	1525 (4.6%)	289 (3.1%)	0.076	308 (3.4%)	289 (3.1%)	0.012
Neuropathy	479 (1.5%)	32 (0.4%)	0.117	27 (0.3%)	32 (0.4%)	0.011
Peripheral angiopathy	712 (2.2%)	36 (0.4%)	0.158	39 (0.4%)	36 (0.4%)	0.005
Poor control	3432 (10.4%)	326 (3.5%)	0.271	327 (3.6%)	326 (3.5%)	0.001
Hypoglycaemia	1735 (5.3%)	72 (0.8%)	0.264	64 (0.7%)	72 (0.8%)	0.009
Insulin resistance	9454 (28.6%)	1117 (12.1%)	0.417	1115 (12.1%)	1117 (12.1%)	0.001
Foot ulcer	608 (1.8%)	52 (0.6%)	0.117	51 (0.6%)	52 (0.6%)	0.003
HbA1c (%)						
<7	3321 (10.0%)	769 (8.4%)	0.058	766 (8.3%)	769 (8.4%)	0.001
7–8.9	13,097 (39.6%)	3426 (37.2%)	0.049	3497 (38.0%)	3426 (37.2%)	0.016
≥9	6765 (20.5%)	1810 (19.7%)	0.019	1777 (19.3%)	1810 (19.7%)	0.009
NA	9888 (29.9%)	3194 (34.7%)	0.103	3159 (34.3%)	3194 (34.7%)	0.008
eGFR (mL/min/1.73m^2^)						
<60	4917 (14.9%)	386 (4.2%)	0.369	353 (3.8%)	386 (4.2%)	0.018
60–89	4547 (13.8%)	1181 (12.8%)	0.027	1274 (13.9%)	1181 (12.8%)	0.030
≥90	4266 (12.9%)	1497 (16.3%)	0.096	1553 (16.9%)	1497 (16.3%)	0.016
NA	19,341 (58.5%)	6135 (66.7%)	0.170	6019 (65.4%)	6135 (66.7%)	0.027
Diagnoses in 3 years prior to initiation (co-morbid conditions)						
Any CVD	14,308 (43.3%)	2483 (27.0%)	0.346	2447 (26.6%)	2483 (27.0%)	0.009
Any cancer	1245 (3.8%)	135 (1.5%)	0.144	138 (1.5%)	135 (1.5%)	0.002
Hypertensive disease	13,097 (39.6%)	2054 (22.3%)	0.380	2041 (22.2%)	2054 (22.3%)	0.003
Hyperlipidaemia	11,717 (35.4%)	1908 (20.7%)	0.331	1917 (20.8%)	1908 (20.7%)	0.002
Medication history of DM drugs 1–365 days prior to initiation						
Number of oral DM drugs						
No records	8051 (24.3%)	2011 (21.9%)	0.059	2212 (24.1%)	2011 (21.9%)	0.052
1	13,331 (40.3%)	3194 (34.7%)	0.116	3039 (33.0%)	3194 (34.7%)	0.036
2	9530 (28.8%)	3532 (38.4%)	0.204	3464 (37.7%)	3532 (38.4%)	0.015
≥3	2159 (6.5%)	462 (5.0%)	0.065	484 (5.3%)	462 (5.0%)	0.011
MET	14,381 (43.5%)	5856 (63.7%)	0.413	5529 (60.1%)	5856 (63.7%)	0.073
SU	21,237 (64.2%)	5162 (56.1%)	0.166	5246 (57.0%)	5162 (56.1%)	0.019
Acarbose	2413 (7.3%)	405 (4.4%)	0.124	444 (4.8%)	405 (4.4%)	0.020
Insulin	5778 (17.5%)	1748 (19.0%)	0.040	1603 (17.4%)	1748 (19.0%)	0.041

DPP4: dipeptidyl peptidase 4; SGLT2: sodium-glucose co-transporter 2; d: standardised difference; SD: standard deviation; SC: Singapore citizen; PR: Singapore permanent resident; SES: socioeconomic status; NA: not applicable; BP: blood pressure; DM: diabetes mellitus; HbA1c: haemoglobin A1c; eGFR: estimated glomerular filtration rate; CVD: cardiovascular disease; MET: metformin; SU: sulfonylureas

Table 7

**Table 7:** Comparison of baseline characteristics in two treatment cohorts before and after PS matching for outcomes measured 31–365 days after initiation in patients of Indian ethnicity

Variables	Unmatched cohorts			Matched cohorts		
	DPP4 inhibitors	SGLT2 inhibitors	d	DPP4 inhibitors	SGLT2 inhibitors	d
	(n = 7326)	(n = 2461)		(n = 2461)	(n = 2461)	
Age (years), mean ± SD	59.8 ± 11.2	55.1 ± 9.4	0.082	55.8 ± 10.3	55.1 ± 9.4	0.013
	n(%)	n(%)		n(%)	n(%)	
Setting of initiation						
Hospitals	3243 (44.3%)	990 (40.2%)	0.082	999 (40.6%)	990 (40.2%)	0.007
Primary care clinics	4083 (55.7%)	1471 (59.8%)	0.082	1462 (59.4%)	1471 (59.8%)	0.007
Year of initiation						
2015	994 (13.6%)	90 (3.7%)	0.359	84 (3.4%)	90 (3.7%)	0.036
2016	1861 (25.4%)	141 (5.7%)	0.564	148 (6.0%)	141 (5.7%)	0.012
2017	2235 (30.5%)	872 (35.4%)	0.105	953 (38.7%)	872 (35.4%)	0.068
2018	2236 (30.5%)	1358 (55.2%)	0.515	1276 (51.9%)	1358 (55.2%)	0.067
Gender (male)	3728 (50.9%)	1307 (53.1%)	0.044	1287 (52.3%)	1307 (53.1%)	0.016
Residence						
SC	6684 (91.2%)	2195 (89.2%)	0.069	2190 (89.0%)	2195 (89.2%)	0.006
PR	642 (8.8%)	266 (10.8%)	0.069	271 (11.0%)	266 (10.8%)	0.006
SES category						
Maximum subsidy	3104 (42.4%)	796 (32.3%)	0.208	800 (32.5%)	796 (32.3%)	0.004
Some subsidy	180 (2.5%)	68 (2.8%)	0.019	67 (2.7%)	68 (2.8%)	0.002
Minimum subsidy	214 (2.9%)	84 (3.4%)	0.028	84 (3.4%)	84 (3.4%)	0
NA	3828 (52.3%)	1513 (61.5%)	0.187	1510 (61.4%)	1513 (61.5%)	0.002
Weight (kilograms)						
<65	1907 (26.0%)	499 (20.3%)	0.137	512 (20.8%)	499 (20.3%)	0.013
65–79	1912 (26.1%)	601 (24.4%)	0.039	616 (25.0%)	601 (24.4%)	0.014
≥80	1207 (16.5%)	550 (22.4%)	0.149	513 (20.9%)	550 (22.4%)	0.036
NA	2300 (31.4%)	811 (33.0%)	0.033	820 (33.3%)	811 (33.0%)	0.008
Cigarette smoking (number of cigarettes per day)						
Non-smoker	2886 (39.4%)	872 (35.4%)	0.082	886 (36.0%)	872 (35.4%)	0.012
1–9	261 (3.6%)	98 (4.0%)	0.022	93 (3.8%)	98 (4.0%)	0.010
≥10	361 (4.9%)	111 (4.5%)	0.020	107 (4.4%)	111 (4.5%)	0.008
NA	3818 (52.1%)	1380 (56.1%)	0.079	1375 (55.9%)	1380 (56.1%)	0.004
Diastolic BP (mmHg)						
<65	1478 (20.2%)	415 (16.9%)	0.085	416 (16.9%)	415 (16.9%)	0.001
65–89	3870 (52.8%)	1309 (53.2%)	0.007	1279 (52.0%)	1309 (53.2%)	0.024
≥90	265 (3.6%)	94 (3.8%)	0.011	112 (4.6%)	94 (3.8%)	0.036
NA	1713 (23.4%)	643 (26.1%)	0.064	654 (26.6%)	643 (26.1%)	0.010
Systolic BP (mmHg)						
<130	2401 (32.8%)	792 (32.2%)	0.013	806 (32.8%)	792 (32.2%)	0.012
130–139	1538 (21.0%)	518 (21.1%)	0.001	502 (20.4%)	518 (21.1%)	0.016
≥140	1674 (22.9%)	508 (20.6%)	0.054	499 (20.3%)	508 (20.6%)	0.009
NA	1713 (23.4%)	643 (26.1%)	0.064	654 (26.6%)	643 (26.1%)	0.010
Duration with diabetes (years)						
0–4	1380 (18.8%)	689 (28.0%)	0.218	659 (26.8%)	689 (28.0%)	0.027
5–9	2224 (30.4%)	636 (25.8%)	0.101	626 (25.4%)	636 (25.8%)	0.009
≥10	3598 (49.1%)	1077 (43.8%)	0.107	1117 (45.4%)	1077 (43.8%)	0.033
NA	124 (1.7%)	59 (2.4%)	0.050	59 (2.4%)	59 (2.4%)	0
Diagnoses for hospitalisation 1–365 days prior to initiation						
Any hospitalisation	2739 (37.4%)	614 (25.0%)	0.271	646 (26.3%)	614 (25.0%)	0.030
DM-kidney complications	776 (10.6%)	64 (2.6%)	0.326	69 (2.8%)	64 (2.6%)	0.012
Retinopathy	411 (5.6%)	102 (4.1%)	0.068	103 (4.2%)	102 (4.1%)	0.003
Neuropathy	129 (1.8%)	21 (0.9%)	0.080	27 (1.1%)	21 (0.9%)	0.025
Peripheral angiopathy	228 (3.1%)	25 (1.0%)	0.147	33 (1.3%)	25 (1.0%)	0.030
Poor control	1071 (14.6%)	201 (8.2%)	0.204	214 (8.7%)	201 (8.2%)	0.019
Hypoglycaemia	403 (5.5%)	45 (1.8%)	0.196	46 (1.9%)	45 (1.8%)	0.003
Insulin resistance	2113 (28.8%)	436 (17.7%)	0.265	448 (18.2%)	436 (17.7%)	0.013
Foot ulcer	187 (2.6%)	28 (1.1%)	0.105	30 (1.2%)	28 (1.1%)	0.007
HbA1c (%)						
<7	561 (7.7%)	195 (7.9%)	0.010	185 (7.5%)	195 (7.9%)	0.015
7–8.9	2510 (34.3%)	750 (30.5%)	0.081	764 (31.0%)	750 (30.5%)	0.012
≥9	1940 (26.5%)	664 (27.0%)	0.011	649 (26.4%)	664 (27.0%)	0.014
NA	2315 (31.6%)	852 (34.6%)	0.064	863 (35.1%)	852 (34.6%)	0.009
eGFR (mL/min/1.73m^2^)						
<60	766 (10.5%)	78 (3.2%)	0.292	77 (3.1%)	78 (3.2%)	0.002
60–89	977 (13.3%)	280 (11.4%)	0.060	295 (12.0%)	280 (11.4%)	0.019
≥90	1424 (19.4%)	514 (20.9%)	0.036	525 (21.3%)	514 (20.9%)	0.011
NA	4159 (56.8%)	1589 (64.6%)	0.160	1564 (63.6%)	1589 (64.6%)	0.021
Diagnoses in 3 years prior to initiation (co-morbid conditions)						
Any CVD	3186 (43.5%)	846 (34.4%)	0.188	833 (33.9%)	846 (34.4%)	0.011
Any cancer	129 (1.8%)	20 (0.8%)	0.084	20 (0.8%)	20 (0.8%)	0
Hypertensive disease	2863 (39.1%)	724 (29.4%)	0.205	712 (28.9%)	724 (29.4%)	0.011
Hyperlipidaemia	2789 (38.1%)	745 (30.3%)	0.165	739 (30.0%)	745 (30.3%)	0.005
Medication history of DM drugs 1–365 days prior to initiation						
Number of oral DM drugs						
No records	1806 (24.7%)	548 (22.3%)	0.056	630 (25.6%)	548 (22.3%)	0.078
1	3031 (41.4%)	921 (37.4%)	0.081	852 (34.6%)	921 (37.4%)	0.058
2	2056 (28.1%)	885 (36.0%)	0.170	873 (35.5%)	885 (36.0%)	0.010
≥3	433 (5.9%)	107 (4.4%)	0.071	106 (4.3%)	107 (4.4%)	0.002
MET	3170 (43.3%)	1546 (62.8%)	0.399	1427 (58.0%)	1546 (62.8%)	0.099
SU	4648 (63.5%)	1304 (53.0%)	0.213	1333 (54.2%)	1304 (53.0%)	0.023
Acarbose	455 (6.2%)	113 (4.6%)	0.072	113 (4.6%)	113 (4.6%)	0
Insulin	1555 (21.2%)	600 (24.4%)	0.075	571 (23.2%)	600 (24.4%)	0.028

DPP4: dipeptidyl peptidase 4; SGLT2: sodium-glucose co-transporter 2; d: standardised difference; SD: standard deviation; SC: Singapore citizen; PR: Singapore permanent resident; SES: socioeconomic status; NA: not applicable; BP: blood pressure; DM: diabetes mellitus; HbA1c: haemoglobin A1c; eGFR: estimated glomerular filtration rate; CVD: cardiovascular disease; MET: metformin; SU: sulfonylureas

Table 8

**Table 8:** Comparison of baseline characteristics in two treatment cohorts before and after PS matching for outcomes measured 31–365 days after initiation in patients of Malay ethnicity

Variables	Unmatched cohorts			Matched cohorts		
	DPP4 inhibitors	SGLT2 inhibitors	d	DPP4 inhibitors	SGLT2 inhibitors	**d**
	(n = 7297)	(n = 2087)		(n = 2087)	(n = 2087)	
Age (years), mean ± SD	60.0 ± 10.8	54.4 ± 9.4	0.097	55.1 ± 10.0	54.4 ± 9.4	0.013
	n(%)	n(%)		n(%)	n(%)	
Setting of initiation						
Hospitals	3296 (45.2%)	767 (36.8%)	0.172	778 (37.3%)	767 (36.8%)	0.011
Primary care clinics	4001 (54.8%)	1320 (63.3%)	0.172	1309 (62.7%)	1320 (63.3%)	0.011
Year of initiation						
2015	948 (13.0%)	48 (2.3%)	0.411	34 (1.6%)	48 (2.3%)	0.095
2016	1731 (23.7%)	96 (4.6%)	0.570	93 (4.5%)	96 (4.6%)	0.007
2017	2332 (32.0%)	738 (35.4%)	0.072	810 (38.8%)	738 (35.4%)	0.071
2018	2286 (31.3%)	1205 (57.7%)	0.551	1150 (55.1%)	1205 (57.7%)	0.053
Gender (male)	3367 (46.1%)	980 (47.0%)	0.016	974 (46.7%)	980 (47.0%)	0.006
Residence						
SC	7091 (97.2%)	2039 (97.7%)	0.033	2041 (97.8%)	2039 (97.7%)	0.007
PR	206 (2.8%)	48 (2.3%)	0.033	46 (2.2%)	48 (2.3%)	0.007
SES category						
Maximum subsidy	3780 (51.8%)	874 (41.9%)	0.200	902 (43.2%)	874 (41.9%)	0.027
Some subsidy	181 (2.5%)	67 (3.2%)	0.044	66 (3.2%)	67 (3.2%)	0.003
Minimum subsidy	119 (1.6%)	43 (2.1%)	0.032	38 (1.8%)	43 (2.1%)	0.017
NA	3217 (44.1%)	1103 (52.9%)	0.176	1081 (51.8%)	1103 (52.9%)	0.021
Weight (kilograms)						
<65	1665 (22.8%)	371 (17.8%)	0.126	390 (18.7%)	371 (17.8%)	0.024
65–79	1840 (25.2%)	505 (24.2%)	0.024	509 (24.4%)	505 (24.2%)	0.004
≥80	1517 (20.8%)	568 (27.2%)	0.151	530 (25.4%)	568 (27.2%)	0.041
NA	2275 (31.2%)	643 (30.8%)	0.008	658 (31.5%)	643 (30.8%)	0.016
Cigarette smoking (number of cigarettes per day)						
Non-smoker	2567 (35.2%)	717 (34.4%)	0.017	717 (34.4%)	717 (34.4%)	0
1–9	261 (3.6%)	85 (4.1%)	0.026	84 (4.0%)	85 (4.1%)	0.003
≥10	434 (6.0%)	174 (8.3%)	0.093	158 (7.6%)	174 (8.3%)	0.028
NA	4035 (55.3%)	1111 (53.2%)	0.042	1128 (54.1%)	1111 (53.2%)	0.016
Diastolic BP (mmHg)						
<65	1377 (18.9%)	349 (16.7%)	0.056	340 (16.3%)	349 (16.7%)	0.012
65–89	4000 (54.8%)	1166 (55.9%)	0.021	1172 (56.2%)	1166 (55.9%)	0.006
≥90	286 (3.9%)	77 (3.7%)	0.012	83 (4.0%)	77 (3.7%)	0.015
NA	1634 (22.4%)	495 (23.7%)	0.032	492 (23.6%)	495 (23.7%)	0.004
Systolic BP (mmHg)						
<130	2189 (30.0%)	617 (29.6%)	0.010	613 (29.4%)	617 (29.6%)	0.004
130–139	1606 (22.0%)	465 (22.3%)	0.007	475 (22.8%)	465 (22.3%)	0.011
≥140	1868 (25.6%)	510 (24.4%)	0.027	507 (24.3%)	510 (24.4%)	0.003
NA	1634 (22.4%)	495 (23.7%)	0.032	492 (23.6%)	495 (23.7%)	0.004
Duration with diabetes (years)						
0–4	1592 (21.8%)	760 (36.4%)	0.326	701 (33.6%)	760 (36.4%)	0.059
5–9	2442 (33.5%)	593 (28.4%)	0.110	599 (28.7%)	593 (28.4%)	0.006
≥10	3202 (43.9%)	704 (33.7%)	0.209	755 (36.2%)	704 (33.7%)	0.051
NA	61 (0.8%)	30 (1.4%)	0.057	32 (1.5%)	30 (1.4%)	0.007
Diagnoses for hospitalisation 1–365 days prior to initiation						
Any hospitalisation	3004 (41.2%)	572 (27.4%)	0.293	608 (29.1%)	572 (27.4%)	0.038
DM-kidney complications	1282 (17.6%)	68 (3.3%)	0.482	53 (2.5%)	68 (3.3%)	0.043
Retinopathy	453 (6.2%)	71 (3.4%)	0.132	76 (3.6%)	71 (3.4%)	0.013
Neuropathy	119 (1.6%)	15 (0.7%)	0.085	20 (1.0%)	15 (0.7%)	0.026
Peripheral angiopathy	250 (3.4%)	12 (0.6%)	0.205	15 (0.7%)	12 (0.6%)	0.019
Poor control	1244 (17.1%)	206 (9.9%)	0.212	214 (10.3%)	206 (9.9%)	0.013
Hypoglycaemia	630 (8.6%)	37 (1.8%)	0.313	40 (1.9%)	37 (1.8%)	0.011
Insulin resistance	2476 (33.9%)	411 (19.7%)	0.326	419 (20.1%)	411 (19.7%)	0.010
Foot ulcer	289 (4.0%)	26 (1.3%)	0.171	26 (1.3%)	26 (1.3%)	0
HbA1c (%)						
<7	678 (9.3%)	183 (8.8%)	0.018	174 (8.3%)	183 (8.8%)	0.015
7–8.9	2454 (33.6%)	654 (31.3%)	0.049	642 (30.8%)	654 (31.3%)	0.013
≥9	1888 (25.9%)	560 (26.8%)	0.022	563 (27.0%)	560 (26.8%)	0.003
NA	2277 (31.2%)	690 (33.1%)	0.040	708 (33.9%)	690 (33.1%)	0.018
eGFR (mL/min/1.73m^2^)						
<60	1106 (15.2%)	71 (3.4%)	0.414	75 (3.6%)	71 (3.4%)	0.010
60–89	880 (12.1%)	318 (15.2%)	0.093	318 (15.2%)	318 (15.2%)	0
≥90	915 (12.5%)	375 (18.0%)	0.151	387 (18.5%)	375 (18.0%)	0.015
NA	4396 (60.2%)	1323 (63.4%)	0.065	1307 (62.6%)	1323 (63.4%)	0.016
Diagnoses in 3 years prior to initiation (co-morbid conditions)						
Any CVD	3638 (49.9%)	754 (36.1%)	0.280	774 (37.1%)	754 (36.1%)	0.020
Any cancer	199 (2.7%)	19 (0.9%)	0.136	23 (1.1%)	19 (0.9%)	0.019
Hypertensive disease	3370 (46.2%)	656 (31.4%)	0.306	680 (32.6%)	656 (31.4%)	0.025
Hyperlipidaemia	3208 (44.0%)	662 (31.7%)	0.254	674 (32.3%)	662 (31.7%)	0.012
Medication history of DM drugs 1–365 days prior to initiation						
Number of oral DM drugs						
No records	1687 (23.1%)	422 (20.2%)	0.070	478 (22.9%)	422 (20.2%)	0.065
1	2838 (38.9%)	682 (32.7%)	0.130	663 (31.8%)	682 (32.7%)	0.019
2	2303 (31.6%)	879 (42.1%)	0.220	845 (40.5%)	879 (42.1%)	0.033
≥3	469 (6.4%)	104 (5.0%)	0.063	101 (4.8%)	104 (5.0%)	0.006
MET	3406 (46.7%)	1398 (67.0%)	0.419	1332 (63.8%)	1398 (67.0%)	0.067
SU	4738 (64.9%)	1220 (58.5%)	0.133	1191 (57.1%)	1220 (58.5%)	0.028
Acarbose	527 (7.2%)	87 (4.2%)	0.132	85 (4.1%)	87 (4.2%)	0.005
Insulin	1658 (22.7%)	456 (21.9%)	0.021	464 (22.2%)	456 (21.9%)	0.009

DPP4: dipeptidyl peptidase 4; SGLT2: sodium-glucose co-transporter 2; d: standardised difference; SD: standard deviation; SC: Singapore citizen; PR: Singapore permanent resident; SES: socioeconomic status; NA: not applicable; BP: blood pressure; DM: diabetes mellitus; HbA1c: haemoglobin A1c; eGFR: estimated glomerular filtration rate; CVD: cardiovascular disease; MET: metformin; SU: sulfonylureas

Table 9

**Table 9:** Comparison of baseline characteristics in two treatment cohorts before and after PS matching for outcomes measured 91–365 days after initiation in patients of Chinese ethnicity

Variables	Unmatched cohorts			Matched cohorts		
	DPP4 inhibitors	SGLT2 inhibitors	d	DPP4 inhibitors	SGLT2 inhibitors	**d**
	(n = 19,335)	(n = 3365)		(n = 3365)	(n = 3365)	
Age (years), mean ± SD	64.4 ± 11.3	57.8 ± 10.7	0.600	58.4 ± 11.1	57.8 ± 10.7	0.052
	n(%)	n(%)		n(%)	n(%)	
Setting of initiation						
Hospitals	7685 (39.8%)	1284 (38.2%)	0.033	1280 (38.0%)	1284 (38.2%)	0.002
Primary care clinics	11,650 (60.3%)	2081 (61.8%)	0.033	2085 (62.0%)	2081 (61.8%)	0.002
Year of initiation						
2015	3526 (18.2%)	180 (5.4%)	0.408	161 (4.8%)	180 (5.4%)	0.026
2016	6766 (35.0%)	391 (11.6%)	0.575	346 (10.3%)	391 (11.6%)	0.043
2017	6537 (33.8%)	1752 (52.1%)	0.375	1863 (55.4%)	1752 (52.1%)	0.066
2018	2506 (13.0%)	1042 (31.0%)	0.446	995 (29.6%)	1042 (31.0%)	0.030
Gender (male)	10,418 (53.9%)	1944 (57.8%)	0.078	1914 (56.9%)	1944 (57.8%)	0.018
Residence						
SC	18,920 (97.9%)	3269 (97.2%)	0.045	3274 (97.3%)	3269 (97.2%)	0.009
PR	415 (2.2%)	96 (2.9%)	0.045	91 (2.7%)	96 (2.9%)	0.009
SES category						
Maximum subsidy	8368 (43.3%)	1002 (29.8%)	0.283	1034 (30.7%)	1002 (29.8%)	0.021
Some subsidy	338 (1.8%)	96 (2.9%)	0.073	88 (2.6%)	96 (2.9%)	0.014
Minimum subsidy	518 (2.7%)	116 (3.5%)	0.045	117 (3.5%)	116 (3.5%)	0.002
NA	10,111 (52.3%)	2151 (63.9%)	0.237	2126 (63.2%)	2151 (63.9%)	0.015
Weight (kilograms)						
<65	6735 (34.8%)	815 (24.2%)	0.234	838 (24.9%)	815 (24.2%)	0.016
65–79	5146 (26.6%)	991 (29.5%)	0.063	976 (29.0%)	991 (29.5%)	0.010
≥80	2631 (13.6%)	757 (22.5%)	0.233	718 (21.3%)	757 (22.5%)	0.028
NA	4823 (24.9%)	802 (23.8%)	0.026	833 (24.8%)	802 (23.8%)	0.021
Cigarette smoking (number of cigarettes per day)						
Non-smoker	6698 (34.6%)	1085 (32.2%)	0.051	1068 (31.7%)	1085 (32.2%)	0.011
1–9	567 (2.9%)	115 (3.4%)	0.028	123 (3.7%)	115 (3.4%)	0.013
≥10	1265 (6.5%)	270 (8.0%)	0.057	265 (7.9%)	270 (8.0%)	0.005
NA	10,805 (55.9%)	1895 (56.3%)	0.009	1909 (56.7%)	1895 (56.3%)	0.008
Diastolic BP (mmHg)						
<65	4690 (24.3%)	687 (20.4%)	0.092	691 (20.5%)	687 (20.4%)	0.003
65–89	10,926 (56.5%)	2027 (60.2%)	0.076	1995 (59.3%)	2027 (60.2%)	0.019
≥90	649 (3.4%)	140 (4.2%)	0.042	153 (4.6%)	140 (4.2%)	0.019
NA	3070 (15.9%)	511 (15.2%)	0.019	526 (15.6%)	511 (15.2%)	0.012
Systolic BP (mmHg)						
<130	6515 (33.7%)	1134 (33.7%)	0	1141 (33.9%)	1134 (33.7%)	0.004
130–139	4598 (23.8%)	885 (26.3%)	0.058	880 (26.2%)	885 (26.3%)	0.003
≥140	5152 (26.7%)	835 (24.8%)	0.042	818 (24.3%)	835 (24.8%)	0.012
NA	3070 (15.9%)	511 (15.2%)	0.019	526 (15.6%)	511 (15.2%)	0.012
Duration with diabetes (years)						
0–4	3645 (18.9%)	926 (27.5%)	0.207	926 (27.5%)	926 (27.5%)	0
5–9	6654 (34.4%)	983 (29.2%)	0.112	919 (27.3%)	983 (29.2%)	0.042
≥10	8779 (45.4%)	1365 (40.6%)	0.098	1430 (42.5%)	1365 (40.6%)	0.039
NA	257 (1.3%)	91 (2.7%)	0.098	90 (2.7%)	91 (2.7%)	0.002
Diagnoses for hospitalisation 1–365 days prior to initiation						
Any hospitalisation	6206 (32.1%)	594 (17.7%)	0.339	627 (18.6%)	594 (17.7%)	0.025
DM-kidney complications	2359 (12.2%)	79 (2.4%)	0.386	74 (2.2%)	79 (2.4%)	0.010
Retinopathy	861 (4.5%)	119 (3.5%)	0.046	121 (3.6%)	119 (3.5%)	0.003
Neuropathy	271 (1.4%)	10 (0.3%)	0.120	12 (0.4%)	10 (0.3%)	0.010
Peripheral angiopathy	366 (1.9%)	18 (0.5%)	0.125	19 (0.6%)	18 (0.5%)	0.004
Poor control	1958 (10.1%)	128 (3.8%)	0.251	147 (4.4%)	128 (3.8%)	0.029
Hypoglycaemia	965 (5.0%)	33 (1.0%)	0.237	25 (0.7%)	33 (1.0%)	0.026
Insulin resistance	5287 (27.3%)	442 (13.1%)	0.359	459 (13.6%)	442 (13.1%)	0.015
Foot ulcer	306 (1.6%)	20 (0.6%)	0.096	24 (0.7%)	20 (0.6%)	0.015
HbA1c (%)						
<7	1536 (7.9%)	371 (11.0%)	0.106	363 (10.8%)	371 (11.0%)	0.008
7–8.9	7677 (39.7%)	1262 (37.5%)	0.045	1285 (38.2%)	1262 (37.5%)	0.014
≥9	4740 (24.5%)	741 (22.0%)	0.059	705 (21.0%)	741 (22.0%)	0.026
NA	5382 (27.8%)	991 (29.5%)	0.036	1012 (30.1%)	991 (29.5%)	0.014
eGFR (mL/min/1.73m^2^)						
<60	2930 (15.2%)	154 (4.6%)	0.360	155 (4.6%)	154 (4.6%)	0.001
60–89	2448 (12.7%)	414 (12.3%)	0.011	428 (12.7%)	414 (12.3%)	0.013
≥90	2456 (12.7%)	574 (17.1%)	0.123	553 (16.4%)	574 (17.1%)	0.017
NA	11,501 (59.5%)	2223 (66.1%)	0.136	2229 (66.2%)	2223 (66.1%)	0.004
Diagnoses in 3 years prior to initiation (co-morbid conditions)						
Any CVD	8240 (42.6%)	997 (29.6%)	0.273	1029 (30.6%)	997 (29.6%)	0.021
Any cancer	612 (3.2%)	60 (1.8%)	0.090	60 (1.8%)	60 (1.8%)	0
Hypertensive disease	7525 (38.9%)	837 (24.9%)	0.305	867 (25.8%)	837 (24.9%)	0.021
Hyperlipidaemia	6811 (35.2%)	768 (22.8%)	0.276	796 (23.7%)	768 (22.8%)	0.020
Medication history of DM drugs 1–365 days prior to initiation						
Number of oral DM drugs						
No records	3779 (19.5%)	554 (16.5%)	0.080	608 (18.1%)	554 (16.5%)	0.043
1	7972 (41.2%)	1305 (38.8%)	0.050	1247 (37.1%)	1305 (38.8%)	0.035
2	5926 (30.7%)	1300 (38.6%)	0.168	1289 (38.3%)	1300 (38.6%)	0.007
≥3	1658 (8.6%)	206 (6.1%)	0.094	221 (6.6%)	206 (6.1%)	0.018
MET	9017 (46.6%)	2193 (65.2%)	0.380	2135 (63.5%)	2193 (65.2%)	0.036
SU	13,453 (69.6%)	2035 (60.5%)	0.192	2054 (61.0%)	2035 (60.5%)	0.011
Acarbose	1815 (9.4%)	180 (5.4%)	0.155	192 (5.7%)	180 (5.4%)	0.016
Insulin	3662 (18.9%)	804 (23.9%)	0.121	771 (22.9%)	804 (23.9%)	0.023

DPP4: dipeptidyl peptidase 4; SGLT2: sodium-glucose co-transporter 2; d: standardised difference; SD: standard deviation; SC: Singapore citizen; PR: Singapore permanent resident; SES: socioeconomic status; NA: not applicable; BP: blood pressure; DM: diabetes mellitus; HbA1c: haemoglobin A1c; eGFR: estimated glomerular filtration rate; CVD: cardiovascular disease; MET: metformin; SU: sulfonylureas

Table 10

**Table 10:** Comparison of baseline characteristics in two treatment cohorts before and after PS matching for outcomes measured 91–365 days after initiation in patients of Indian ethnicity

Variables	Unmatched cohorts			Matched cohorts		
	DPP4 inhibitors	SGLT2 inhibitors	d	DPP4 inhibitors	SGLT2 inhibitors	d
	(n = 4181)	(n = 905)		(n = 905)	(n = 905)	
Age (years), mean ± SD	60.0 ± 10.9	55.2 ± 9.4	0.477	56.0 ± 10.0	55.2 ± 9.4	0.086
	n(%)	n(%)		n(%)	n(%)	
Setting of initiation						
Hospitals	1810 (43.3%)	406 (44.9%)	0.026	401 (44.3%)	406 (44.9%)	0.011
Primary care clinics	2371 (56.7%)	499 (55.1%)	0.026	504 (55.7%)	499 (55.1%)	0.011
Year of initiation						
2015	736 (17.6%)	73 (8.1%)	0.243	70 (7.7%)	73 (8.1%)	0.013
2016	1575 (37.7%)	117 (12.9%)	0.497	102 (11.3%)	117 (12.9%)	0.051
2017	1367 (32.7%)	415 (45.9%)	0.222	466 (51.5%)	415 (45.9%)	0.113
2018	503 (12.0%)	300 (33.2%)	0.412	267 (29.5%)	300 (33.2%)	0.079
Gender (male)	2090 (50.0%)	473 (52.3%)	0.038	474 (52.4%)	473 (52.3%)	0.002
Residence						
SC	3860 (92.3%)	827 (91.4%)	0.028	834 (92.2%)	827 (91.4%)	0.028
PR	321 (7.7%)	78 (8.6%)	0.028	71 (7.9%)	78 (8.6%)	0.028
SES category						
Maximum subsidy	1860 (44.5%)	281 (31.1%)	0.231	295 (32.6%)	281 (31.1%)	0.033
Some subsidy	104 (2.5%)	33 (3.7%)	0.055	29 (3.2%)	33 (3.7%)	0.025
Minimum subsidy	88 (2.1%)	30 (3.3%)	0.060	24 (2.7%)	30 (3.3%)	0.039
NA	2129 (50.9%)	561 (62.0%)	0.185	557 (61.6%)	561 (62.0%)	0.009
Weight (kilograms)						
<65	1155 (27.6%)	202 (22.3%)	0.102	211 (23.3%)	202 (22.3%)	0.024
65–79	1212 (29.0%)	253 (28.0%)	0.019	244 (27.0%)	253 (28.0%)	0.022
≥80	809 (19.4%)	234 (25.9%)	0.127	240 (26.5%)	234 (25.9%)	0.015
NA	1005 (24.0%)	216 (23.9%)	0.003	210 (23.2%)	216 (23.9%)	0.016
Cigarette smoking (number of cigarettes per day)						
Non-smoker	1668 (39.9%)	322 (35.6%)	0.073	331 (36.6%)	322 (35.6%)	0.021
1–9	174 (4.2%)	43 (4.8%)	0.023	40 (4.4%)	43 (4.8%)	0.016
≥10	227 (5.4%)	46 (5.1%)	0.013	53 (5.9%)	46 (5.1%)	0.034
NA	2112 (50.5%)	494 (54.6%)	0.067	481 (53.2%)	494 (54.6%)	0.029
Diastolic BP (mmHg)						
<65	940 (22.5%)	186 (20.6%)	0.039	193 (21.3%)	186 (20.6%)	0.019
65–89	2452 (58.7%)	531 (58.7%)	0	533 (58.9%)	531 (58.7%)	0.005
≥90	150 (3.6%)	40 (4.4%)	0.034	38 (4.2%)	40 (4.4%)	0.011
NA	639 (15.3%)	148 (16.4%)	0.024	141 (15.6%)	148 (16.4%)	0.021
Systolic BP (mmHg)						
<130	1519 (36.3%)	335 (37.0%)	0.012	348 (38.5%)	335 (37.0%)	0.030
130–139	979 (23.4%)	210 (23.2%)	0.004	200 (22.1%)	210 (23.2%)	0.026
≥140	1044 (25.0%)	212 (23.4%)	0.030	216 (23.9%)	212 (23.4%)	0.010
NA	639 (15.3%)	148 (16.4%)	0.024	141 (15.6%)	148 (16.4%)	0.021
Duration with diabetes (years)						
0–4	679 (16.2%)	219 (24.2%)	0.161	198 (21.9%)	219 (24.2%)	0.055
5–9	1424 (34.1%)	253 (28.0%)	0.109	242 (26.7%)	253 (28.0%)	0.027
≥10	2021 (48.3%)	410 (45.3%)	0.050	442 (48.8%)	410 (45.3%)	0.071
NA	57 (1.4%)	23 (2.5%)	0.068	23 (2.5%)	23 (2.5%)	0
Diagnoses for hospitalisation 1–365 days prior to initiation						
Any hospitalisation	1522 (36.4%)	243 (26.9%)	0.171	258 (28.5%)	243 (26.9%)	0.037
DM-kidney complications	442 (10.6%)	31 (3.4%)	0.242	34 (3.8%)	31 (3.4%)	0.018
Retinopathy	229 (5.5%)	42 (4.6%)	0.032	39 (4.3%)	42 (4.6%)	0.016
Neuropathy	72 (1.7%)	11 (1.2%)	0.035	12 (1.3%)	11 (1.2%)	0.010
Peripheral angiopathy	118 (2.8%)	12 (1.3%)	0.089	15 (1.7%)	12 (1.3%)	0.027
Poor control	616 (14.7%)	89 (9.8%)	0.125	95 (10.5%)	89 (9.8%)	0.022
Hypoglycaemia	216 (5.2%)	17 (1.9%)	0.153	21 (2.3%)	17 (1.9%)	0.031
Insulin resistance	1208 (28.9%)	167 (18.5%)	0.206	168 (18.6%)	167 (18.5%)	0.003
Foot ulcer	100 (2.4%)	10 (1.1%)	0.084	12 (1.3%)	10 (1.1%)	0.021
HbA1c (%)						
<7	250 (6.0%)	106 (11.7%)	0.162	97 (10.7%)	106 (11.7%)	0.031
7–8.9	1447 (34.6%)	287 (31.7%)	0.051	278 (30.7%)	287 (31.7%)	0.021
≥9	1250 (29.9%)	220 (24.3%)	0.104	243 (26.9%)	220 (24.3%)	0.058
NA	1234 (29.5%)	292 (32.3%)	0.049	287 (31.7%)	292 (32.3%)	0.012
eGFR (mL/min/1.73m^2^)						
<60	456 (10.9%)	27 (3.0%)	0.272	22 (2.4%)	27 (3.0%)	0.034
60–89	523 (12.5%)	109 (12.0%)	0.012	113 (12.5%)	109 (12.0%)	0.014
≥90	892 (21.3%)	196 (21.7%)	0.007	210 (23.2%)	196 (21.7%)	0.037
NA	2310 (55.3%)	573 (63.3%)	0.135	560 (61.9%)	573 (63.3%)	0.030
Diagnoses in 3 years prior to initiation (co-morbid conditions)						
Any CVD	1882 (45.0%)	329 (36.4%)	0.146	316 (34.9%)	329 (36.4%)	0.030
Any cancer	71 (1.7%)	10 (1.1%)	0.043	10 (1.1%)	10 (1.1%)	0
Hypertensive disease	1696 (40.6%)	285 (31.5%)	0.157	281 (31.1%)	285 (31.5%)	0.009
Hyperlipidaemia	1648 (39.4%)	295 (32.6%)	0.117	299 (33.0%)	295 (32.6%)	0.009
Medication history of DM drugs 1–365 days prior to initiation						
Number of oral DM drugs						
No records	840 (20.1%)	178 (19.7%)	0.009	174 (19.2%)	178 (19.7%)	0.011
1	1776 (42.5%)	361 (39.9%)	0.043	338 (37.4%)	361 (39.9%)	0.052
2	1239 (29.6%)	310 (34.3%)	0.081	330 (36.5%)	310 (34.3%)	0.046
≥3	326 (7.8%)	56 (6.2%)	0.052	63 (7.0%)	56 (6.2%)	0.031
MET	1906 (45.6%)	570 (63.0%)	0.291	556 (61.4%)	570 (63.0%)	0.032
SU	2883 (69.0%)	492 (54.4%)	0.247	536 (59.2%)	492 (54.4%)	0.098
Acarbose	345 (8.3%)	59 (6.5%)	0.055	66 (7.3%)	59 (6.5%)	0.030
Insulin	1002 (24.0%)	271 (29.9%)	0.110	257 (28.4%)	271 (29.9%)	0.034

DPP4: dipeptidyl peptidase 4; SGLT2: sodium-glucose co-transporter 2; d: standardised difference; SD: standard deviation; SC: Singapore citizen; PR: Singapore permanent resident; SES: socioeconomic status; NA: not applicable; BP: blood pressure; DM: diabetes mellitus; HbA1c: haemoglobin A1c; eGFR: estimated glomerular filtration rate; CVD: cardiovascular disease; MET: metformin; SU: sulfonylureas.

Table 11

**Table 11:** Comparison of baseline characteristics in two treatment cohorts before and after PS matching for outcomes measured 91–365 days after initiation in patients of Malay ethnicity

Variables	Unmatched cohorts			Matched cohorts		
	DPP4 inhibitors	SGLT2 inhibitors	d	DPP4 inhibitors	SGLT2 inhibitors	d
	(n = 4065)	(n = 745)		(n = 745)	(n = 745)	
Age (years), mean ± SD	59.9 ± 10.4	54.9 ± 9.0	0.520	55.2 ± 10.1	54.9 ± 9.0	0.031
	n(%)	n(%)		n(%)	n(%)	
Setting of initiation						
Hospitals	1670 (41.1%)	285 (38.3%)	0.058	308 (41.3%)	285 (38.3%)	0.063
Primary care clinics	2395 (58.9%)	460 (61.7%)	0.058	437 (58.7%)	460 (61.7%)	0.063
Year of initiation						
2015	726 (17.9%)	32 (4.3%)	0.442	26 (3.5%)	32 (4.3%)	0.042
2016	1432 (35.2%)	81 (10.9%)	0.604	80 (10.7%)	81 (10.9%)	0.004
2017	1414 (34.8%)	372 (49.9%)	0.310	410 (55.0%)	372 (49.9%)	0.102
2018	493 (12.1%)	260 (34.9%)	0.557	229 (30.7%)	260 (34.9%)	0.089
Gender (male)	1862 (45.8%)	338 (45.4%)	0.009	334 (44.8%)	338 (45.4%)	0.011
Residence						
SC	3960 (97.4%)	733 (98.4%)	0.068	730 (98.0%)	733 (98.4%)	0.030
PR	105 (2.6%)	12 (1.6%)	0.068	15 (2.0%)	12 (1.6%)	0.030
SES category						
Maximum subsidy	2108 (51.9%)	329 (44.2%)	0.155	322 (43.2%)	329 (44.2%)	0.019
Some subsidy	104 (2.6%)	19 (2.6%)	0.001	21 (2.8%)	19 (2.6%)	0.017
Minimum subsidy	69 (1.7%)	14 (1.9%)	0.014	16 (2.2%)	14 (1.9%)	0.019
NA	1784 (43.9%)	383 (51.4%)	0.151	386 (51.8%)	383 (51.4%)	0.008
Weight (kilograms)						
<65	919 (22.6%)	117 (15.7%)	0.176	122 (16.4%)	117 (15.7%)	0.019
65–79	1135 (27.9%)	217 (29.1%)	0.027	213 (28.6%)	217 (29.1%)	0.012
≥80	959 (23.6%)	249 (33.4%)	0.219	227 (30.5%)	249 (33.4%)	0.063
NA	1052 (25.9%)	162 (21.7%)	0.097	183 (24.6%)	162 (21.7%)	0.067
Cigarette smoking (number of cigarettes per day)						
Non-smoker	1351 (33.2%)	248 (33.3%)	0.001	251 (33.7%)	248 (33.3%)	0.008
1–9	166 (4.1%)	33 (4.4%)	0.017	32 (4.3%)	33 (4.4%)	0.006
≥10	273 (6.7%)	83 (11.1%)	0.155	76 (10.2%)	83 (11.1%)	0.030
NA	2275 (56.0%)	381 (51.1%)	0.097	386 (51.8%)	381 (51.1%)	0.013
Diastolic BP (mmHg)						
<65	825 (20.3%)	147 (19.7%)	0.014	144 (19.3%)	147 (19.7%)	0.010
65–89	2405 (59.2%)	473 (63.5%)	0.089	457 (61.3%)	473 (63.5%)	0.044
≥90	164 (4.0%)	27 (3.6%)	0.021	33 (4.4%)	27 (3.6%)	0.041
NA	671 (16.5%)	98 (13.2%)	0.095	111 (14.9%)	98 (13.2%)	0.050
Systolic BP (mmHg)						
<130	1264 (31.1%)	254 (34.1%)	0.064	240 (32.2%)	254 (34.1%)	0.040
130–139	1006 (24.8%)	176 (23.6%)	0.026	173 (23.2%)	176 (23.6%)	0.009
≥140	1124 (27.7%)	217 (29.1%)	0.033	221 (29.7%)	217 (29.1%)	0.012
NA	671 (16.5%)	98 (13.2%)	0.095	111 (14.9%)	98 (13.2%)	0.050
Duration with diabetes (years)						
0–4	819 (20.2%)	249 (33.4%)	0.303	236 (31.7%)	249 (33.4%)	0.037
5–9	1540 (37.9%)	207 (27.8%)	0.216	219 (29.4%)	207 (27.8%)	0.036
10	1676 (41.2%)	273 (36.6%)	0.094	277 (37.2%)	273 (36.6%)	0.011
NA	30 (0.7%)	16 (2.2%)	0.118	13 (1.7%)	16 (2.2%)	0.030
Diagnoses for hospitalisation 1–365 days prior to initiation						
Any hospitalisation	1544 (38.0%)	199 (26.7%)	0.243	214 (28.7%)	199 (26.7%)	0.045
DM-kidney complications	638 (15.7%)	21 (2.8%)	0.455	20 (2.7%)	21 (2.8%)	0.009
Retinopathy	235 (5.8%)	25 (3.4%)	0.116	22 (3.0%)	25 (3.4%)	0.023
Neuropathy	60 (1.5%)	8 (1.1%)	0.037	9 (1.2%)	8 (1.1%)	0.013
Peripheral angiopathy	114 (2.8%)	1 (0.1%)	0.224	0	1 (0.1%)	0.051
Poor control	666 (16.4%)	74 (9.9%)	0.192	80 (10.7%)	74 (9.9%)	0.027
Hypoglycaemia	314 (7.7%)	15 (2.0%)	0.268	14 (1.9%)	15 (2.0%)	0.009
Insulin resistance	1288 (31.7%)	154 (20.7%)	0.253	164 (22.0%)	154 (20.7%)	0.033
Foot ulcer	131 (3.2%)	12 (1.6%)	0.105	14 (1.9%)	12 (1.6%)	0.021
HbA1c (%)						
<7	281 (6.9%)	93 (12.5%)	0.189	88 (11.8%)	93 (12.5%)	0.021
7–8.9	1390 (34.2%)	236 (31.7%)	0.053	232 (31.1%)	236 (31.7%)	0.012
≥9	1174 (28.9%)	207 (27.8%)	0.024	200 (26.9%)	207 (27.8%)	0.021
NA	1220 (30.0%)	209 (28.1%)	0.043	225 (30.2%)	209 (28.1%)	0.047
eGFR (mL/min/1.73m^2^)						
<60	655 (16.1%)	27 (3.6%)	0.428	25 (3.4%)	27 (3.6%)	0.014
60–89	439 (10.8%)	121 (16.2%)	0.160	128 (17.2%)	121 (16.2%)	0.025
≥90	503 (12.4%)	137 (18.4%)	0.167	133 (17.9%)	137 (18.4%)	0.014
NA	2468 (60.7%)	460 (61.7%)	0.021	459 (61.6%)	460 (61.7%)	0.003
Diagnoses in 3 years prior to initiation (co-morbid conditions)						
Any CVD	1967 (48.4%)	274 (36.8%)	0.236	283 (38.0%)	274 (36.8%)	0.025
Any cancer	97 (2.4%)	6 (0.8%)	0.126	5 (0.7%)	6 (0.8%)	0.016
Hypertensive disease	1826 (44.9%)	248 (33.3%)	0.240	255 (34.2%)	248 (33.3%)	0.020
Hyperlipidaemia	1762 (43.4%)	241 (32.4%)	0.228	242 (32.5%)	241 (32.4%)	0.003
Medication history of DM drugs 1–365 days prior to initiation						
Number of oral DM drugs						
No records	762 (18.8%)	122 (16.4%)	0.062	136 (18.3%)	122 (16.4%)	0.050
1	1560 (38.4%)	271 (36.4%)	0.041	257 (34.5%)	271 (36.4%)	0.039
2	1389 (34.2%)	304 (40.8%)	0.137	304 (40.8%)	304 (40.8%)	0
≥3	354 (8.7%)	48 (6.4%)	0.086	48 (6.4%)	48 (6.4%)	0
MET	2046 (50.3%)	493 (66.2%)	0.325	465 (62.4%)	493 (66.2%)	0.078
SU	2863 (70.4%)	466 (62.6%)	0.168	475 (63.8%)	466 (62.6%)	0.025
Acarbose	382 (9.4%)	40 (5.4%)	0.155	45 (6.0%)	40 (5.4%)	0.029
Insulin	925 (22.8%)	183 (24.6%)	0.042	172 (23.1%)	183 (24.6%)	0.035

DPP4: dipeptidyl peptidase 4; SGLT2: sodium-glucose co-transporter 2; d: standardised difference; SD: standard deviation; SC: Singapore citizen; PR: Singapore permanent resident; SES: socioeconomic status; NA: not applicable; BP: blood pressure; DM: diabetes mellitus; HbA1c: haemoglobin A1c; eGFR: estimated glomerular filtration rate; CVD: cardiovascular disease; MET: metformin; SU: sulfonylureas

## Abbreviations

HbA1c: haemoglobin A1c
eGFR: estimated glomerular filtration rate
BP: blood pressure
SGLT2: sodium-glucose co-transporter 2
DPP4: dipeptidyl peptidase 4
MET: metformin
SU: sulfonylureas
T2DM: type 2 diabetes mellitus
T1DM: type 1 diabetes mellitus
DM: diabetes mellitus
CKD: chronic kidney disease
DKA: diabetic ketoacidosis
CVD: cardiovascular disease
HF: heart failure
UTI: urinary tract infection
SC: Singapore citizen
PR: Singapore permanent resident
SES: socioeconomic status
MOH: Ministry of Health, Singapore
ICD-10 AM: International Classification of Diseases, Tenth Revision Australian Modification
RR: risk ratio
CI: confidence interval
PS: propensity score
d: standardised difference
SD: standard deviation
MD: mean difference
HR: hazard ratio
OR: odds ratio
RCTs: randomised controlled trials
CVD-REAL: Comparative Effectiveness of Cardiovascular Outcomes in New Users of Sodium-Glucose Cotransporter-2 Inhibitors
CANVAS: Canagliflozin Cardiovascular Assessment Study
EMPA-REG OUTCOME: Empagliflozin Cardiovascular Outcome Event Trial in Type 2 Diabetes Mellitus Patients–Removing Excess Glucose
UK: United Kingdom
US: United States
NA: not applicable
